# Single-cell roadmap of human gonadal development

**DOI:** 10.1038/s41586-022-04918-4

**Published:** 2022-07-06

**Authors:** Luz Garcia-Alonso, Valentina Lorenzi, Cecilia Icoresi Mazzeo, João Pedro Alves-Lopes, Kenny Roberts, Carmen Sancho-Serra, Justin Engelbert, Magda Marečková, Wolfram H. Gruhn, Rachel A. Botting, Tong Li, Berta Crespo, Stijn van Dongen, Vladimir Yu Kiselev, Elena Prigmore, Mary Herbert, Ashley Moffett, Alain Chédotal, Omer Ali Bayraktar, Azim Surani, Muzlifah Haniffa, Roser Vento-Tormo

**Affiliations:** 1 Wellcome Sanger Institute, Cambridge, UK; 2 Wellcome Trust/Cancer Research UK Gurdon Institute, University of Cambridge, Cambridge, UK; 3 Physiology, Development and Neuroscience Department, University of Cambridge, Cambridge, UK; 4 Biosciences Institute, Newcastle University, Newcastle upon Tyne, UK; 5 Nuffield Department of Women’s and Reproductive Health, University of Oxford, Oxford, UK; 6 Great Ormond Street Institute of Child Health, University College London, London, UK; 7 University of Cambridge Centre for Trophoblast Research, Department of Pathology, University of Cambridge, Cambridge, UK; 8 Sorbonne Université, INSERM, CNRS, Institut de la Vision, Paris, France; 9 Wellcome-MRC Cambridge Stem Cell Institute, Jeffrey Cheah Biomedical Centre, Cambridge, UK

**Keywords:** Cell biology, Differentiation

## Abstract

Gonadal development is a complex process that involves sex determination followed by divergent maturation into either testes or ovaries^1^. Historically, limited tissue accessibility, a lack of reliable in vitro models and critical differences between humans and mice have hampered our knowledge of human gonadogenesis, despite its importance in gonadal conditions and infertility. Here, we generated a comprehensive map of first- and second-trimester human gonads using a combination of single-cell and spatial transcriptomics, chromatin accessibility assays and fluorescent microscopy. We extracted human-specific regulatory programmes that control the development of germline and somatic cell lineages by profiling equivalent developmental stages in mice. In both species, we define the somatic cell states present at the time of sex specification, including the bipotent early supporting population that, in males, upregulates the testis-determining factor *SRY* and sPAX8s, a gonadal lineage located at the gonadal–mesonephric interface. In females, we resolve the cellular and molecular events that give rise to the first and second waves of granulosa cells that compartmentalize the developing ovary to modulate germ cell differentiation. In males, we identify human *SIGLEC15*^+^ and *TREM2*^*+*^ fetal testicular macrophages, which signal to somatic cells outside and inside the developing testis cords, respectively. This study provides a comprehensive spatiotemporal map of human and mouse gonadal differentiation, which can guide in vitro gonadogenesis.

## Abstract (web summary)

This study provides a comprehensive spatiotemporal map of human and mouse gonadal differentiation, using a combination of single-cell and spatial transcriptomics, chromatin accessibility assays and fluorescent microscopy, which can guide in vitro gonadogenesis.

## Main

In humans, the undifferentiated bipotent gonads, which emerge on the ventral surface of the mesonephros, commit to ovarian or testicular fate. Around 6 weeks after conception (postconceptional weeks; PCW)^1^, gonadal somatic cells expressing *SRY*, the Y-linked testis-determining factor, differentiate into Sertoli cells (testicular supporting cells) or, in its absence, into pregranulosa cells (preGCs; ovarian supporting cells)^2^. Sertoli cells and preGCs coordinate the differentiation of the remaining sex-specific gonadal somatic (for example, interstitial) and germ cell lineages^3^. In males, primordial germ cells (PGCs), the gamete precursors, differentiate into prespermatogonia, forming cord-like structures with Sertoli cells and entering mitotic arrest. In females, PGCs differentiate into oogonia, which enter an asynchronous transition from mitosis to meiosis. Later in development, granulosa cells surround primary oocytes to form primordial follicles, remaining quiescent until puberty^4^.

Here, we used single-cell multiomics and spatial methods to disentangle the cellular and molecular programmes that mediate human gonadal development in space and time. We uncover previously uncharacterized cellular heterogeneity in the somatic lineage, with relevance for gonadal conditions that have their origin during development, such as differences in sex development. In addition, we generated mouse single-cell transcriptomics data to contextualize our human findings with the mouse counterpart, facilitating translational research between these species.

## Human–mouse gonadal atlas

**Fig. 1:**
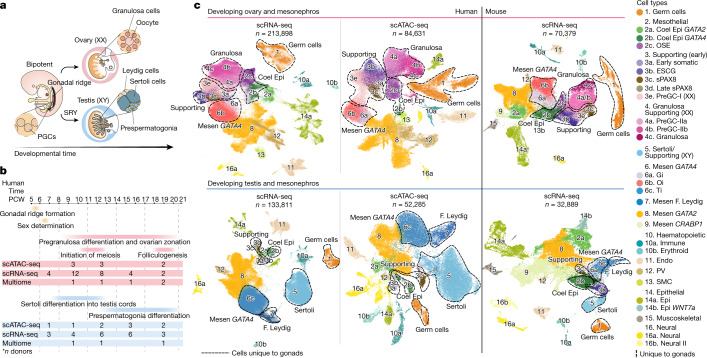
Human–mouse harmonized single-cell atlases of gonadal and extragonadal tissue. **a**, Schematic illustration of gonadal development showing the main structures of the XX and XY gonads. **b**, Diagram summarizing the stage and sex composition of our sample cohort along with main events occurring during gonadogenesis. **c**, Top shows the UMAP of cell lineages (colour) in the human female scRNA-seq (*n* = 213,898), human female scATAC-seq (*n* = 84,631) and mouse female scRNA-seq (*n* = 70,379) datasets. Bottom shows UMAP projections of cell lineages (colour) in the human male scRNA-seq (*n* = 133,811), human male scATAC-seq (*n* = 52,285) and mouse male scRNA-seq (*n* = 32,889) datasets. Clusters for mesothelial, supporting and gonadal mesenchymal *LHX9*^*+*^ cells were defined in an independent per-lineage reanalysis and projected onto this dataset (Fig. 3). Dashed lines outline the cell populations unique to the gonads. Doublets and low-quality control cells were removed. CoelEpi, coelomic epithelium; Endo, endothelial; Epi, epithelial; F. Leydig, fetal Leydig; Gi, gonadal interstitial; Mesen, mesenchymal; Oi, ovarian interstitial; OSE, ovarian surface epithelium; preGC, pregranulosa cells; PV, perivascular; sPAX8, supporting *PAX8 *^+^; Ti, testicular interstitial; SMC, smooth muscle cell.

**Extended Data Fig. 1:**
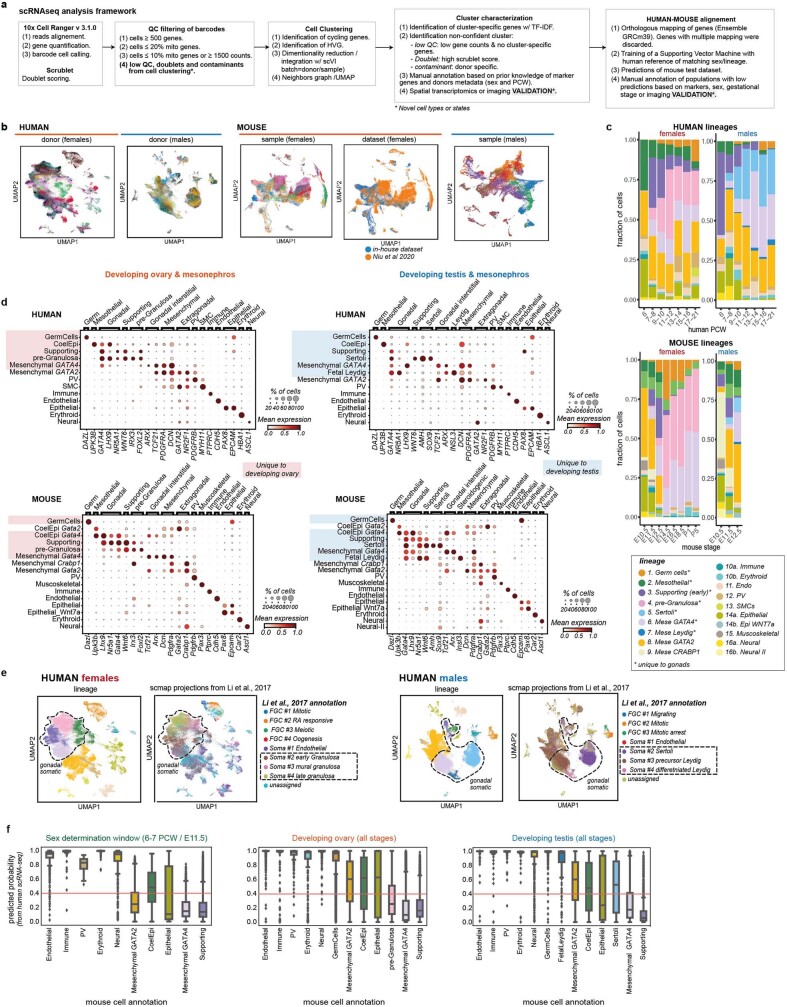
Quality control of scRNA-seq data of the human developing ovaries and testes. **a**, Schematic representation of the computational workflow used to analyse scRNA-seq data. **b**, UMAP (uniform manifold approximation and projection) of the male and female human (left) and mouse (right) scRNA-seq datasets labelled by donor and sample. Dots from the same donor or sample share a colour. For female mouse scRNA-seq data, an additional UMAP is coloured by the study of origin. **c**, Barplot showing the proportions of human (top) and mouse (bottom) cells profiled with scRNA-seq coloured by lineage and classified by sex and developmental stage (indicated in post-conceptional weeks (PCW) or embryonic (E) / postnatal (P) days). **d**, Dot plots showing the variance-scaled, log-transformed expression of genes (X-axis) characteristic of the main lineages (Y-axis) detected in male and female human (top) and mouse (bottom) scRNA-seq datasets. Top-layer groups marker genes by categories. Lineages unique to developing ovaries and testes are highlighted with "*". **e**, Predicted cell annotations from Li *et al*. 2017 scRNA-seq analysis of human gonads on our human scRNA-seq dataset. Labels were transferred using *scmap* separately for females (left) and males (right), with a cutoff of 0.5. Cells that do not pass the 0.5 cutoff are labelled as “unassigned”. Colour legend for the main lineages match those in Extended Data Fig. 1c. **f**, Boxplot showing the predicted probabilities of human cell types transferred with a Support Vector Machine (SVM) model onto manually annotated mouse cell types around the time of sex determination (n = 29,297 cells; left) for both females and males, or considering all developmental stages combined for ovaries (n = 70,379 cells; middle) and testes (n = ; 32,889; right) separately. The box extends from the lower to upper quartile values of the data, with a line at the median. The whiskers extend from the box to show the range of the data. Flyer points are those past the end of the whiskers. CoelEpi = coelomic epithelium; E = embryonic day; Endo = endothelial; Epi = epithelial; FGC = fetal germ cells; P = postnatal day; PCW = post-conceptional weeks; SMC = smooth muscle cells; Soma = somatic; PV = perivascular; Mese = mesenchymal. Source data

**Extended Data Fig. 2:**
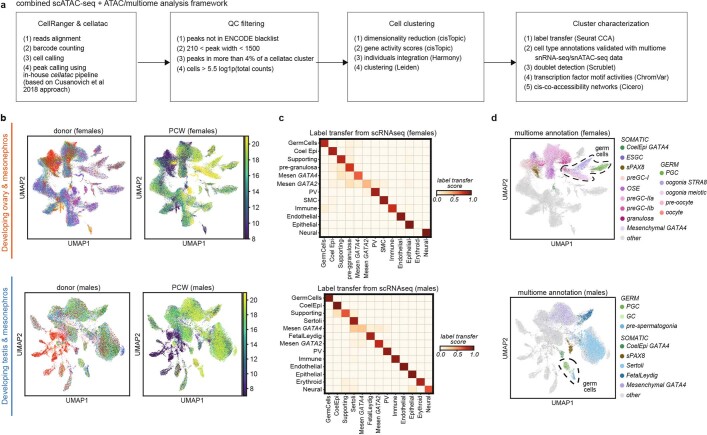
Analysis of the chromatin accessibility landscape of the human developing ovaries and testes. **a**, Schematic representation of the computational workflow used to analyse human scATAC-seq data. **b**, UMAP (uniform manifold approximation and projection) of female (top) and male (bottom) human scATAC-seq datasets labelled by donor and post-conceptional weeks (PCW). Dots from the same donor share a colour. **c**, Heatmap reporting label transfer scores from human scRNA-seq to scATAC-seq female (left) and male (right) data of matched individuals. Colour intensity corresponds to the average of the label transfer scores for all cells in each annotated cell type. **d**, UMAP projections of female (left) and male (right) human scATAC-seq datasets labelled by the cell lineage identified in snRNA-seq data from the cell-coupled snRNA-seq/snATAC-seq profiling. Only gonadal tissue was included in the combined snRNA-seq/scATAC-seq assay. CoelEpi = coelomic epithelium; ESGC = early supporting gonadal cells; GC = germ cells; Mesen = mesenchymal; OSE = ovarian surface epithelium; PCW = post-conceptional weeks; PGC = primordial germ cells; preGC = pregranulosa cells; PV = perivascular; SMC = smooth muscle cell; sPAX8 = supporting PAX8.

We profiled human gonadal and adjacent extragonadal tissue from the first and second trimesters of gestation (6–21 PCW), covering stages of sex determination and differentiation into ovaries and testes (female *n* = 33, male *n* = 22; Fig. 1a,b). We used several single-cell genomics methods: (1) single-cell RNA sequencing (scRNA-seq); (2) single-cell accessible chromatin sequencing (scATAC-seq) and (3) combined single-nucleus RNA and ATAC sequencing (snRNA-seq/scATAC-seq) to profile 347,709, 96,174 and 40,742 cells, respectively (Fig. 1b and Supplementary Tables 1–3). We also generated single-cell transcriptomes of corresponding mouse tissue around the time of sex determination, that is, at embryonic days (E) 10.5, 11.5 and 12.5 (63,929 cells), and integrated them with a previously published dataset covering later gestational stages (E11.5 to postnatal day (P) 5)^5^ (Supplementary Table 1). Male and female samples were analysed separately and cell annotation was assigned on the basis of the expression of known markers and label transfer from scRNA-seq to scATAC-seq^6^ (Fig. 1c, Extended Data Figs. 1a–d and 2a–d and Supplementary Note 1). The abundant number of cells profiled in our study allowed us to resolve new somatic cell states, which were not defined in a previous human gonadal scRNA-seq study^7^ (Extended Data Fig. 1e).

**Extended Data Fig. 3:**
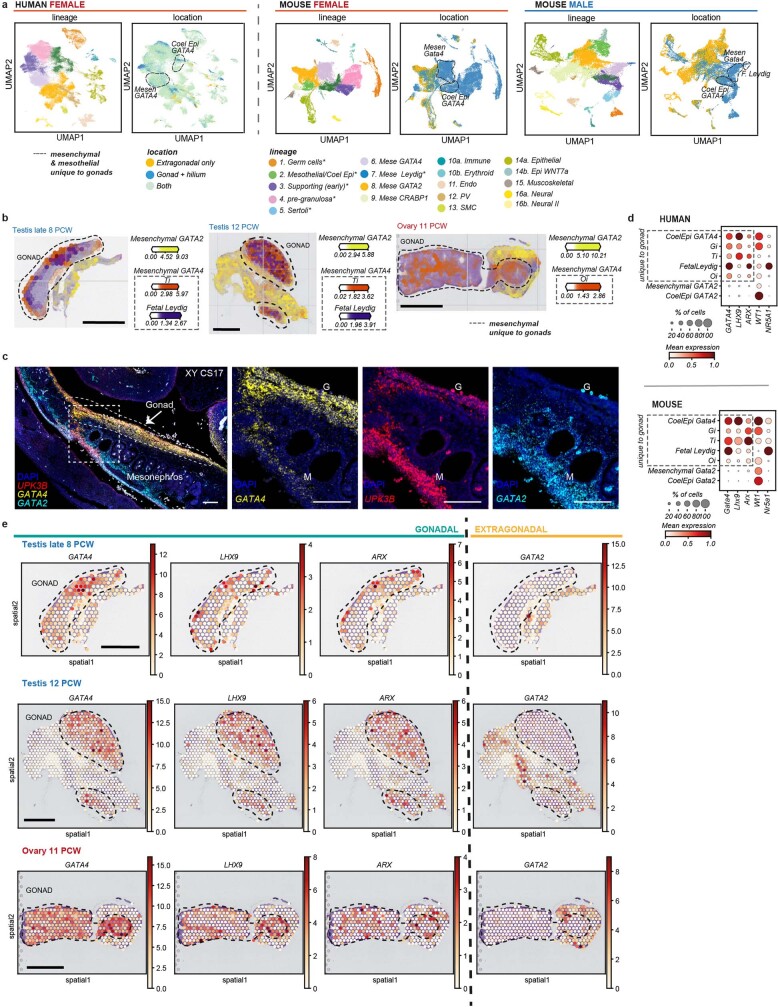
Gonadal and extragonadal location of mesenchymal and mesothelial cells. **a**, UMAP (uniform manifold approximation and projection) of human (left) and mouse (right) scRNA-seq datasets labelled by cell lineage and tissue location. **b**, Spatial mapping of mesenchymal cell types from the human scRNA-seq dataset to a spatial transcriptomics slide of a late 8 post-conceptional weeks (PCW) testis, a 12 PCW testis and an 11 PCW ovary with cell2location. Estimated abundance for cell types (colour intensity) contributed by each mesenchymal subpopulation to each Visium spot (colour) shown over the H&E images. Scale bars = 1 mm; n = 2. **c**, High-resolution imaging of a representative gonadal section (sagittal) of a human XY fetal gonad (Carnegie stage, (CS)17). Intensity proportional to smFISH signal for *UPK3B* (red, coelomic epithelium), *GATA4* (yellow, intra-gonadal) and *GATA2* (cyan, extra-gonadal); n = 5. The white dashed rectangles (left) highlight the enlarged sample region (right). Scale bars = 100 µm (left) and 10 µm in magnified regions (right). **d**, Dot plot showing the variance-scaled, log-transformed expression of transcription factors (TFs) with mutually exclusive expression in gonadal and extragonadal mesenchymal and mesothelial cells in human and mouse scRNA-seq data. **e**, Spatial plot showing the variance-scaled, log-transformed expression of TF with mutually exclusive expression in gonadal and extragonadal mesenchymal cells over the H&E images of a late 8 PCW testis, a 12 PCW testis and an 11 PCW ovary; n = 2. Scale bars = 1 mm. CoelEpi = coelomic epithelium; Endo = endothelial; Epi = epithelial; G = gonad; Gi = gonadal interstitial; M = mesonephros; Mese = mesenchymal; Oi = ovarian interstitial; PV = perivascular cell; SMC = smooth muscle cell; Ti = testicular interstitial.

To locate cells in the profiled tissues we (1) generated spatial transcriptomics data using Visium and multiplexed single-molecule fluorescent in situ hybridization (smFISH), and (2) isolated the gonad and extragonadal tissue by microdissection to profile each separately. Germ (*DAZL*^*+*^) and supporting (*GATA4*^+^, *WNT6*^+^) cells are present exclusively within the gonads, whereas other cell types, including coelomic epithelial (*UPK3B*^+^) and mesenchymal (*PDGFRA*^+^) cells, are present in both the gonads and the mesonephros (extragonadal tissue) (Fig. 1c and Extended Data Fig. 3a). In both humans and mice, expression of the transcription factors (TFs) *GATA4*, *LHX9* and *ARX* is a hallmark of the gonadal coelomic epithelium and mesenchymal cells, whereas *GATA2* expression is restricted to the mesonephros and other extragonadal tissue (Extended Data Fig. 3b–e and Supplementary Note 1).

We find strong correspondence between the transcriptomic signatures of the primary cell lineages in humans and mice, using a support vector machine (SVM) classifier trained on the human cells (Extended Data Fig. 1f and Supplementary Note 2). Notable exceptions with low similarity (median prediction probability < 0.4) are the early supporting and gonadal mesenchymal lineages in both sexes, and pregranulosa cells in females, which suggests a divergence in the development of somatic cell lineages between humans and mice.

## TFs modulating germ cell differentiation

**Extended Data Fig. 4:**
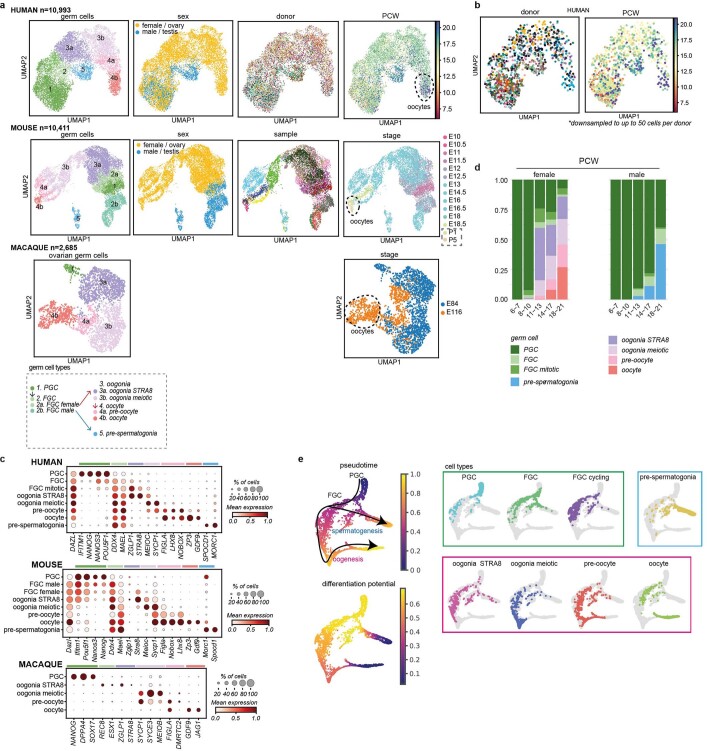
Characterisation of germ cell states. **a**, UMAP (uniform manifold approximation and projection) of germ cell in the human (top; n = 10,993), mouse (middle; n = 10,411) and external macaque (bottom: n = 2,685) scRNA-seq datasets labelled by germ cell states, sex, donor/sample identity and developmental stage indicated in post-conceptional weeks (PCW) or embryonic (E) / postnatal (P) days). Doublets and low QC cells removed. **b**, Downsampled UMAP for human germ cells to account for up to 50 cells per donor (colour) for confirmatory visualisation. **c**, Dot plots showing the variance-scaled, log-transformed expression of genes characteristic of fetal oogenesis in the human (top), mouse (middle) and macaque (bottom) germ cells scRNA-seq data. **d**, Relative proportion of human germ cell states (colour) profiled with scRNA-seq, classified by sex and developmental stage. **e**, t-SNE (t-distributed stochastic neighbour embedding) projection of scRNA-seq data of human germ cells coloured by Palantir pseudo-time and probability of cells to progress from the PGC status. Germ cells are downsampled to account for 500 cells for each germ cell state. E = embryonic day; FGC = fetal germ cells; P = postnatal day; PCW = post-conceptional weeks; PGC = primordial germ cells.

**Extended Data Fig. 5:**
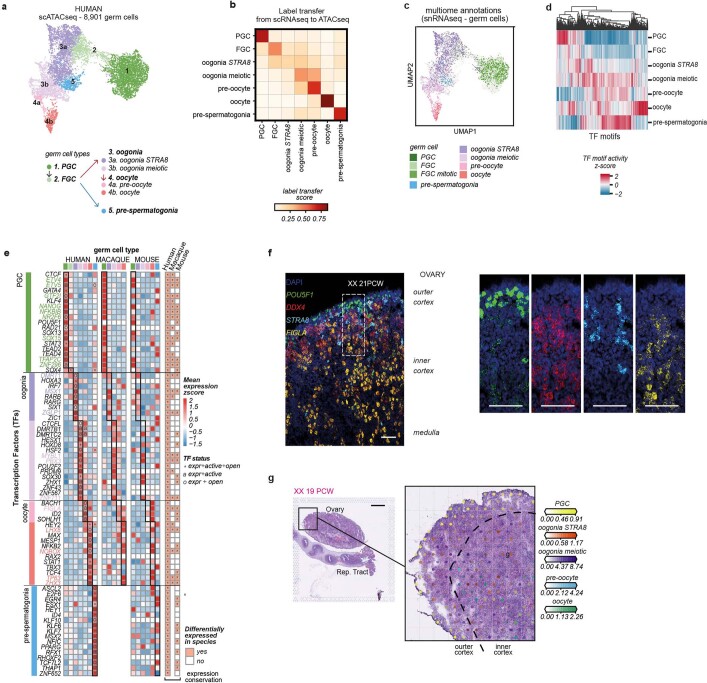
Cross species TF comparison of germ cells. **a**, UMAP of germ cell states (colour) in the human scATAC-seq (n = 8,901) dataset. Doublets and low QC cells removed. **b**, Heatmap reporting label transfer scores from human scRNA-seq to scATAC-seq germ cell data of matched individuals. **c**, UMAP of germ cells in the human scATAC-seq dataset labelled by cell state identified in snRNA-seq data from the cell-coupled snRNA-seq/snATAC-seq profiling. **d**, Hierarchical clustering of transcription factor (TF) binding activity scores in each human germ cell type estimated from scATAC-seq data. **e**, Heatmaps showing the expression of human-relevant transcription factors (TF) in human, macaque and mouse germ cells. Colour proportional to scaled log-transformed expression. For human germ cells only: “o” = TF whose binding motifs are differentially accessible (i.e. TF can bind their potential targets); “a” = TF whose targets are differentially expressed (i.e. differentially activated TF); and asterisk (*) = TF that meets both “o” and “a” conditions. Conservation heatmap (right) highlights significant overexpression (log2-fold change > 0 and FDR < 0.05) in each species. TFs whose upregulation is conserved across species are highlighted with bold/coloured labels. **f**, High-resolution imaging of a representative transverse section of a human ovary at 21 post-conceptional weeks (PCW), with intensity proportional to smFISH signal for *POU5F1* (green, primordial germ cells), *DDX4* (red, fetal germ cells), *STRA8* (cyan, pre-meiotic germ cells) and *FIGLA* (yellow, oocytes); n = 4. The white dashed rectangle highlights the enlarged gonadal region. Scale bars = 100 µm. **g**, *cell2location* estimated cell abundance (colour intensity) contributed by each germ cell to each Visium spot (colour) shown over the H&E image of a 19 PCW ovary; n = 2. Scale bars = 1 mm. E = embryonic day; Expr = expressed, FGC = fetal germ cells; P = postnatal day; PCW = post-conceptional weeks; PGC = primordial germ cells.

PGCs colonize the human gonads at roughly 3–5 PCW^8,9^ and, guided by the male and female supporting cells, start their differentiation into either prespermatogonia or oogonia at roughly 6 PCW. To compare the differentiation of human germ cells with that of other mammals, we integrated our human and mouse gonadal germ cells with more scRNA-seq gonadal germ cells datasets from mouse and macaque^5,10,11^ (Extended Data Fig. 4a–d, Supplementary Table 4 and Supplementary Note 3). We used trajectory reconstruction methods to trace the differentiation of human PGCs into prespermatogonia and oocytes (Extended Data Fig. 4e), and investigated the TF programmes that mediate these transitions. We prioritized those TFs that were differentially expressed and active in humans using both the transcriptome and open chromatin data (Extended Data Fig. 5a–d) and compared their expression dynamics between humans, macaques and mice (Extended Data Fig. 5e and Supplementary Table 5). We identify *GATA4* as a primate-specific TF upregulated in PGCs. In all species analysed, we find *SOX4* is active in PGCs and prespermatogonia but downregulated during oogenesis. In addition, the transition from PGCs to prespermatogonia involves the activation of *EGR4*, *KLF6* and *KLF7*. Fetal oocyte differentiation is more complex than its male counterpart: it involves meiosis initiation and a spatial trajectory, with PGCs restricted to the outer cortex and cells migrating towards the medulla as they differentiate^7^ (Extended Data Fig. 5f,g). Before meiosis initiation, coinciding with a premeiotic *STRA8* surge, we find the activation of *ZGLP1*, the oogenic TF recently described in mice^12^ (Extended Data Fig. 5e). At this premeiotic stage, human oogonia also upregulate the *ZIC1* factor, which is involved in retinoic acid production^13^ that is necessary for meiosis induction. After entering meiosis, oogonia activate *DMRTC2* and *ZNF711*, previously described in mice, together with another DMRT member, *DMRTB1*, which is analogously upregulated in macaque and mouse oogonia. Furthermore, there is upregulation of HOX factors (for example, *HOXA3*, *HOXD8*) and cofactors (for example, *PBX3*) in distinct oogonia stages. In oocytes, we find activation of *TP63* and *ZHX3*, with conserved expression dynamics in macaques and mice.

## Somatic cells during sex determination

**Fig. 2:**
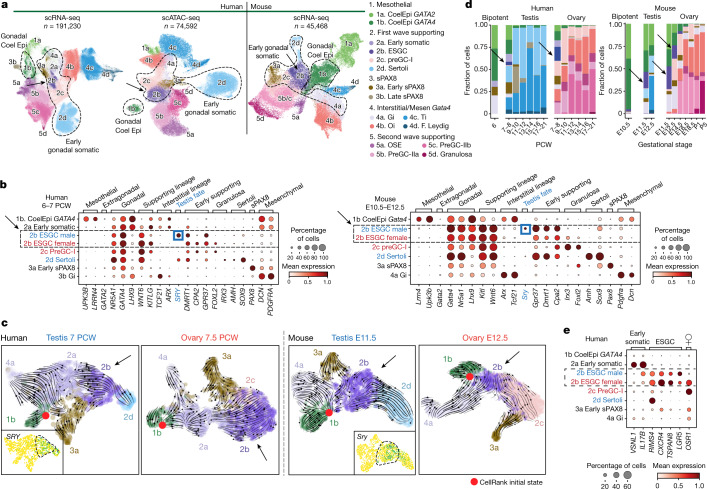
New gonadal somatic cells during sex determination in humans and mice. **a**, UMAP of somatic cell states (colour) in the human scRNA-seq (*n* = 191,230), human scATAC-seq (*n* = 74,592) and mouse scRNA-seq (*n* = 45,468) datasets. Doublets and low-quality control cells were removed. **b**, Dot plots show the variance-scaled, log-transformed expression of genes (*x*-axis) characteristic of the first wave of somatic cells (*y*-axis) in humans and mice. **c**, UMAP of somatic cells overlaid with RNA velocity maps in two humans (7 PCW testis; 7.5 PCW ovary) and two mice (E11.5 testis, E12.5 ovary) gonadal samples, analysed independently. **d**, Relative proportions of human and mouse somatic cell states (colour) profiled with scRNA-seq, classified by sex and developmental stage. Black arrows highlight the ESGCs. **e**, Dot plot showing the variance-scaled, log-transformed expression of human-specific early somatic and ESGC markers (*x*-axis) in the first wave of human supporting cells (*y*-axis). CoelEpi, coelomic epithelium; Gi, gonadal interstitial; Oi, ovarian interstitial; preGC, pregranulosa cells; sPAX8, supporting PAX8; Ti, testicular interstitial.

**Extended Data Fig. 6:**
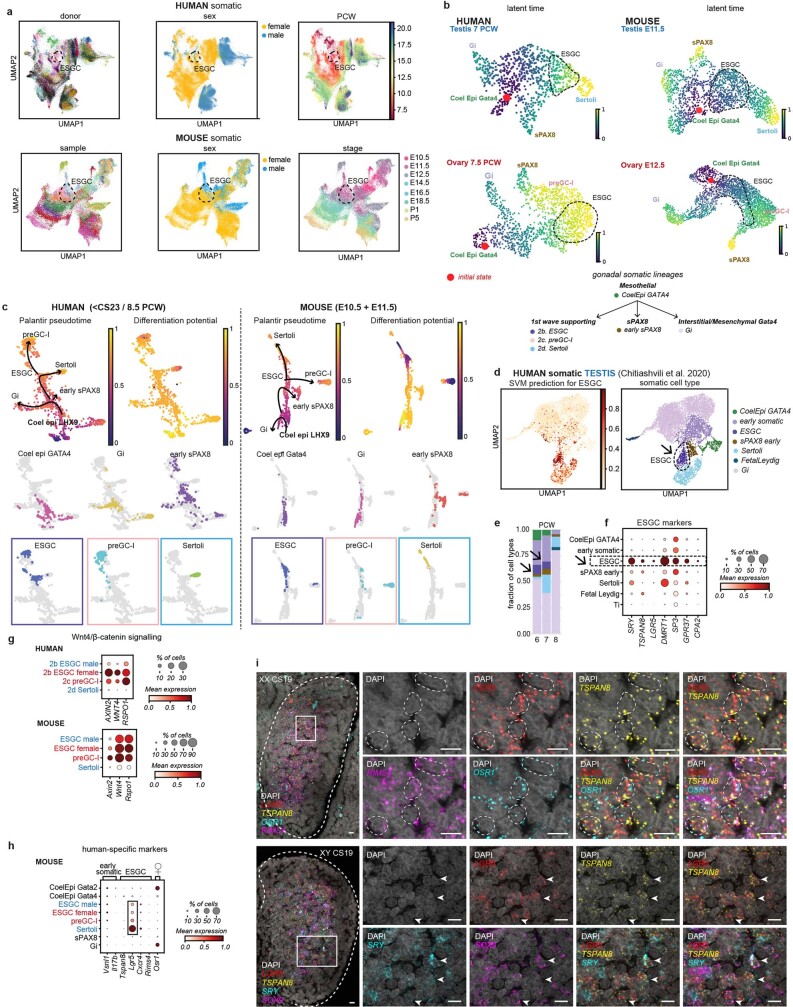
Human-mouse comparison and trajectory inference of early gonadal somatic cells. **a**, UMAP (uniform manifold approximation and projection) of gonadal somatic cells in the human (top) and mouse (bottom) scRNA-seq datasets coloured by sample of origin, sex and developmental stage (indicated in post-conceptional weeks (PCW) or embryonic (E) / postnatal (P) day). Dots from the same donor or sample share a colour. **b**, UMAP projections of the fate probabilities of each cell ending up in one of the terminal states (scRNA-seq). Coloured symbols indicate the initial and terminal cell states predicted by CellRank. Top UMAPs depict two human (7 post-conceptional weeks, PCW testis; 7.5 PCW ovary) gonadal samples while bottom UMAPs depict two mouse (E11.5 testis and E12.5 ovary) gonadal samples, analysed independently. **c**, t-SNE (t-distributed stochastic neighbour embedding) projection of somatic cells coloured by Palantir pseudo-time and probability of cells to progress from the gonadal coelomic epithelium *GATA4+* in humans between 6-8.5 PCW (left) and mice at E10.5-E11.5 (right). Somatic cells are downsampled to account for 150 cells for each cell state in each sex in both species. **d**, (left) UMAP projections of the predicted probability of ESGC from our dataset onto Guo et al., 2021 somatic cells manifold using a Support Vector Machine (SVM) classifier. (right) UMAP projections on the validation dataset of human fetal testis, re-analysed from Guo et al., 2021, labelled by somatic cell state. **e**, Barplot showing the proportions of somatic cells in the Guo et al., 2021 dataset coloured by cell state and classified by PCW. **f**, Dot plots showing the variance-scaled, log-transformed expression of genes characteristic of human ESGC in the Guo et al., 2021 dataset of human fetal testis. **g**, Dot plot showing the variance-scaled, log-transformed expression of genes in the WNT4/RSPO1 pathway in ESGC (split in male and female), preGC-I and Sertoli cells in the human (top) and mouse (bottom) scRNA-seq dataset. **h**, Dot plots showing the variance-scaled, log-transformed expression of human-specific markers of ESGC in the mouse scRNA-seq dataset. **i**, High-resolution, imaging of representative human gonadal sections with intensity proportional to smFISH signal for RNA markers. (top) Carnegie stage 19 (CS19) ovary stained for *LGR5* (red, ESGC), *TSPAN8* (yellow, ESGC), *RIMS4* (magenta, 1st wave somatic cells), *OSR1* (cyan, preGC-I). The white dashed line outlines the ovary; the white dashed rectangle highlights the enlarged gonadal region. ESGCs nuclei have been marked with dashed circles. (bottom) CS19 testis stained for *LGR5* (red, ESGC), *TSPAN8* (yellow, ESGC), *SRY* (magenta, ESGC), *SOX9* (cyan, Sertoli). The white dashed line outlines the testis. The white dashed rectangle highlights the enlarged gonadal region. White arrows in the magnified areas mark ESGC nuclei; n = 2. Scale bars = 100 µm and 10 µm in magnified regions. CoelEpi = coelomic epithelium; E = embryonic day; ESGC = early supporting gonadal cells; Gi = gonadal interstitial; P = postnatal day; PCW = post-conceptional week; preGC = pre-granulosa cells; sPAX8 = supporting PAX8; Ti = testicular interstitial.

The coelomic epithelium in the gonadal ridge is the primary source of gonadal somatic cells^3,14^. Using trajectory inference methods, we identify the bipotent early supporting gonadal cells (ESGCs), connecting the *GATA4*^*+*^ coelomic epithelium with either Sertoli cells or the first wave of preGCs (preGC-I) in both humans and mice (Fig. 2a–c, Extended Data Fig. 6a–c and Supplementary Note 4). ESGCs appear transiently in the early gonads (roughly 6–8 PCW in humans and E11.5 in mice) and, in males, are the first gonadal somatic cells to express the testis-determining factor *SRY*, which is required for Sertoli cell commitment^2^ (Fig. 2b,d, Extended Data Fig. 6d–f and Supplementary Table 6). Thus, ESGCs are the bipotent precursors that give rise to the sex-specific supporting cells in the early gonad.

We identify a core set of genes with conserved expression dynamics between humans and mice as the *GATA4*^*+*^ coelomic epithelial cells, which differentiate into the first wave of supporting cells. In humans, the coelomic epithelium and ESGCs are connected through an early somatic cell population that downregulates mesothelial markers (*UPK3B*^−^, *LRRN4*^*−*^), upregulates supporting lineage markers (*WNT6*^*+*^) and shares TFs with undifferentiated gonadal interstitial (Gi) cells (*ARX*^*+*^, *TCF21*^*+*^; Fig. 2b). Next, human and mouse ESGCs downregulate *LHX9* and interstitial TFs (*ARX*^−^, *TCF21*^*−*^) while further upregulating the supporting lineage marker *WNT6*. ESGCs also upregulate *GPR37* and *DMRT1*, the latter being essential for testis development^15^. Male ESGCs are *SRY*^+^ and initiate the downregulation of the pro-ovarian RSPO1/WNT4–β-catenin pathway (*WNT4*, *RSPO1*, *AXIN2*; Extended Data Fig. 6g). Accordingly, the expression of *FOXL2*, which is essential for ovarian fate^16,17^, can already be detected in female ESGCs at this stage (Fig. 2b).

Human ESGCs upregulate stem-cell markers (*LGR5*^*+*^, *TSPAN8*^*+*^; Fig. 2e and Extended Data Fig. 6h). *LGR5* shows a different expression pattern between humans and mice: in humans, *LGR5* is specific to ESGCs; in mice, *Lgr5* is upregulated during the second wave of pregranulosa and Sertoli cell formation, with basal expression in ESGCs (Extended Data Fig. 6h). We detected the expression of *TSPAN8* only in human ESGCs (Fig. 2e and Extended Data Fig. 6h). Human female ESGCs also upregulate *OSR1*, characteristic of preGC-I, which is notably absent in mice (Fig. 2e and Extended Data Fig. 6h). Using a combination of these markers, we located ESGCs in the developing human testes and ovaries by multiplexed smFISH (Extended Data Fig. 6i). At early 8 PCW (Carnegie stage (CS)19–CS20), ESGCs (*TSPAN8*^*+*^, *LGR5*^*+*^) reside in the ovarian medulla together with the preGC-I (*OSR1*^*+*^) in females, or the developing testis cords with early Sertoli cells (*SOX9*^*+*^, *LGR5*^*−*^) in males.

## *PAX8*^*+*^ cells define gonadal boundaries

**Extended Data Fig. 7:**
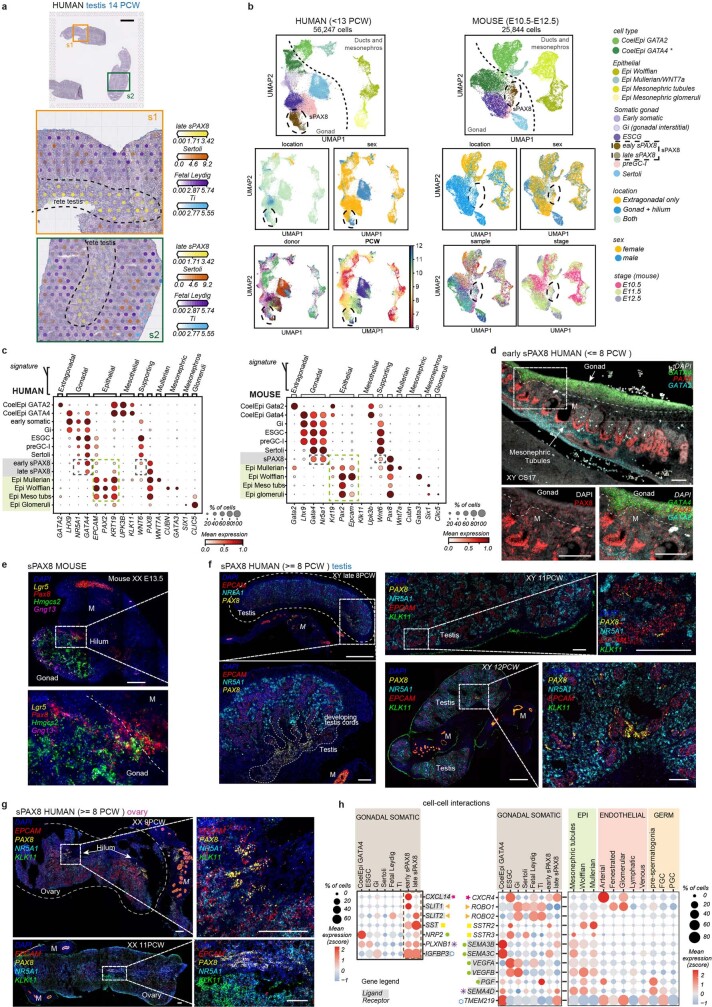
Gonadal supporting *PAX8+* cells define gonadal boundaries. **a**, Spatial mapping of somatic cell types from the scRNA-seq human dataset to three consecutive spatial transcriptomics slides of a 14 PCW testis using *cell2location*. Estimated abundance for cell types (colour intensity) contributed by each cell population to each spot (colour) shown over the H&E image; n = 3. Scale bars = 1 mm. **b**, UMAP (uniform manifold approximation and projection) showing mesothelial, first wave supporting and epithelial cells in human first trimester (left) and mouse embryonic day (E) E10.5-E12.5 (right) scRNA-seq data labelled by cell type, location of the tissue, sex, donor or sample and post-conceptional weeks (PCW) or stage. **c**, Dot plot showing the variance-scaled, log-transformed expression of genes characteristic of the mesothelial, supporting and epithelial subpopulations in human first trimester (top) and mouse E10.5-E12.5 (bottom) scRNA-seq data. **d**, High-resolution large-area imaging of representative gonadal section (sagittal) of a human fetal testis (7PCW, Carnegie Stage CS17) with intensity proportional to smFISH signal for *GATA4* (green, gonadal), *PAX8* (red, sPAX8 population) and *GATA2* (cyan, extragonadal); n = 5. This sample is also shown in Extended Data Fig. 3c. **e**, High-resolution large-area imaging of a representative section of a mouse fetal ovary (E13.5) with intensity proportional to smFISH signal for *Lgr5* (yellow, cortical pre-granulosa), *Pax8* (red, sPAX8), *Hmgcs2* (green, medullary pre-granulosa) and *Gng13* (magenta, cortical pre-granulosa); n = 2. **f**, High-resolution large-area imaging of representative sections of three human fetal testes (sagittal late 8, transverse 11 and transverse 12 PCW; n = 3) with intensity proportional to smFISH signal for *PAX8* (yellow, sPAX8 population), *NR5A1* (cyan, interstitial Fetal Leydig), *EPCAM* (red, low in supporting cells, high in epithelial cells of the reproductive tubules) and *KLK11* (green; coelomic epithelium). **g**, High-resolution large-area imaging of representative sections of two human fetal ovaries (9 and 11 PCW; n = 2) with intensity proportional to smFISH signal for the same panel in “f”. **h**, (left) Dot plot showing the scaled log-transformed expression of upregulated genes coding for sPAX8 ligands or receptor proteins in the supporting testis cells. (right) Dot plots showing the scaled log-transformed expression of genes coding for cognate ligand or receptor proteins in the supporting epithelial, endothelial and germ cells. Interacting partners (i.e., with binding specificity) are linked with a matching symbol. CoelEpi = coelomic epithelium; E = embryonic day; Epi = epithelial; ESGC = early supporting gonadal cells; FGC = fetal germ cells; Gi = gonadal interstitial; M = mesonephros; PCW = post-conceptional week; PGC = primordial germ cells; preGC = pre-granulosa cells; sPAX8 = supporting PAX8; Ti = testicular interstitial. For all smFISH panels, unless otherwise specified, white dashed rectangles highlight gonadal regions magnified; scale bars = 100 µm and 10 µm in magnified regions.

The gonadal–mesonephric interface is a site of extensive tissue remodelling during early gonadogenesis, regulating cell migration, vascularization and formation of the rete testis, a network of tubules that connects the testis cords with the reproductive ducts^18,19^. We define a supporting-like *PAX8*^+^ population (sPAX8s) expressing gonadal (*GATA4*^+^, *LHX9*^+^ and *NR5A1*^+^) and supporting (*WNT6*^+^) markers that emerges with the first wave of supporting cells in humans and mice (Fig. 2a–d and Supplementary Note 5). sPAX8s are located at the site where the rete testis will form in the testis, as shown by Visium (Extended Data Fig. 7a), and are clearly distinct from epithelial cells in the Mullerian and Wolffian ducts, as shown by their low expression of epithelial markers (*EPCAM*^*low*^, *KRT19*^*low*^) and their independent clustering when analysed with epithelial cells (Extended Data Fig. 7b,c).

**Fig. 3:**
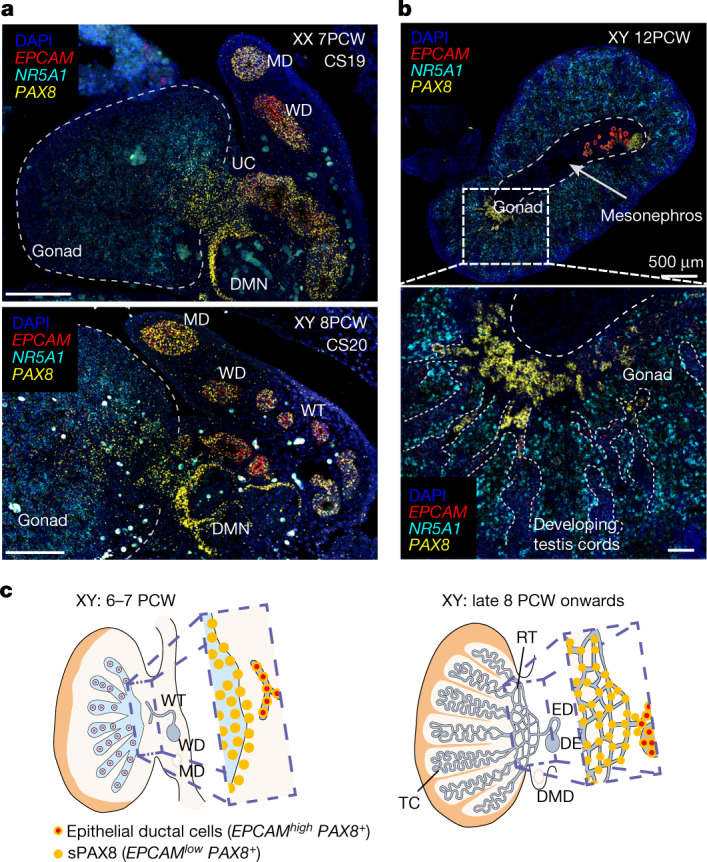
Supporting-like *PAX8*^+^ (sPAX8) gonadal lineage forms the rete testis. **a**, High-resolution large-area imaging of representative gonadal sections (transverse) of a human ovary (7 PCW, CS19; top) and testis (8 PCW, CS20; bottom), with intensity proportional to smFISH signal for *EPCAM* (red, epithelial), *NR5A1* (cyan, gonadal somatic) and *PAX8* (yellow, sPAX8 and epithelial) (*n* = 2); red blood cells appear as bright autofluorescent cells. **b**, High-resolution large-area imaging of representative gonadal sections of one human testis (12 PCW, transverse section), with intensity proportional to smFISH signal for *EPCAM* (red, epithelial), *NR5A1* (cyan, gonadal somatic) and *PAX8* (yellow, sPAX8 and epithelial) (*n* = 2). White dashed rectangles highlight enlarged gonadal regions with *PAX8*^*high*^ *EPCAM*^*low*^ expression. **c**, Schematic representation of sPAX8 cells in the human testis at two developmental stages. DE, ductus epididymidis; DMD, degenerating Müllerian duct; DMN, degenerating mesonephric nephron; ED, efferent ductule; MD, Mullerian duct; RT, rete testis; TC, testis cords; UC, urogenital connection; WD, Wolffian duct; WT, Wolffian tubules; scale bars, 100 µm unless otherwise specified.

Our in-depth analysis of human samples covering a broad developmental window allowed us to distinguish two subsets of sPAX8s in humans: early and late sPAX8s. Early sPAX8s are sexually undifferentiated cells enriched at roughly 6–8 PCW in both sexes (Fig. 2d). Staining the gonads with smFISH shows that early sPAX8s (*PAX8*^* +*^, *EPCAM*^ low^) are found inside the gonad, at the gonadal–mesonephric interface, until 8 PCW (CS17 to CS20) (Fig. 3a and Extended Data Fig. 7d). We also found this population at a similar location in mice (Extended Data Fig. 7e). Late sPAX8s (*PAX8*^*+*^, *EPCAM*^*low*^) are present only in males from late 8 PCW (Fig. 2d). smFISH analyses detected sPAX8s at the poles of the developing testis cords where the rete testis will develop, in agreement with the Visium data (Fig. 3b and Extended Data Fig. 7a,f). In developing human ovaries after 8 PCW, only a few sPAX8s were found near the hilum (Extended Data Fig. 7g), in keeping with the presence of a rudimentary rete ovarii that degenerates at later stages^20^.

Both sPAX8 subsets show a unique transcriptional pattern of axon guidance factors, suggesting they have a structural and supporting role. In humans, early sPAX8s express *CXCL14*, and its receptor *CXCR4* is expressed by endothelial and supporting cells, suggesting a chemotactic role for these populations (Extended Data Fig. 7h). Male late sPAX8s express *NRP2*, the receptor for *VEGF* and *SEMA3B/C*, which are upregulated by epithelial cells. sPAX8s distinctively express somatostatin (*SST*) and *IGFBP3*, whose receptors are upregulated in various cells, including supporting, epithelial, endothelial and coelomic epithelial cells. Together, these data suggest that sPAX8s are a gonadal supporting-like cell lineage in mammals that mediate the formation of the rete testis and rete ovarii.

## The second wave of pregranulosa cells

**Fig. 4:**
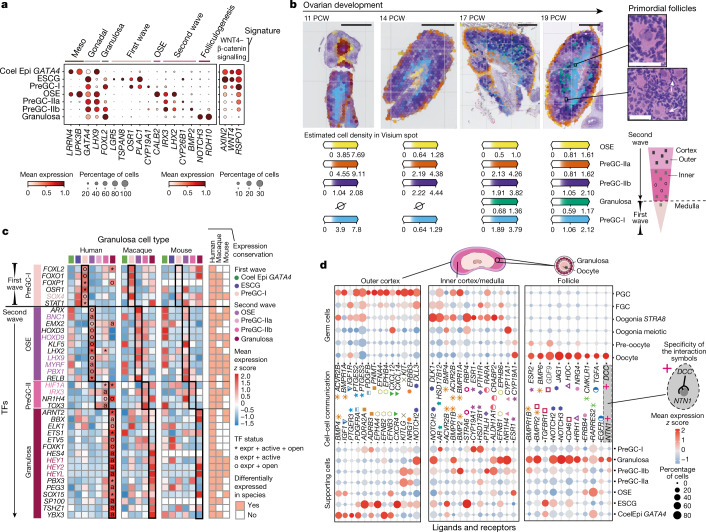
Transcriptional, spatiotemporal and paracrine signatures of human pregranulosa cells. **a**, Dot plots show the variance-scaled, log-transformed expression of genes (*x*-axis) characteristic of ovarian supporting cells (*y*-axis) in human scRNA-seq data. Top layer groups marker genes by categories. **b**, Spatial mapping of granulosa cell types from the scRNA-seq human dataset to spatial transcriptomics slide of 11, 14, 17 and 19 PCW ovaries using cell2location; *n* = 2. Estimated cell abundance (colour intensity) for OSE, preGC-I, preGC-IIa, preGC-IIb and developing granulosa cells (colour) in each Visium spot shown over the haematoxylin and eosin (H&E) images. The black rectangles highlight enlarged ovarian regions with forming follicles (top right). Schematic representation of the spatial organization of pregranulosa cell states in the human ovary (bottom right). Scale bars 1 mm (left) and 50 µm in magnified regions (right). **c**, Heatmaps showing expression of selected TFs across human, macaque and mouse ovarian supporting cells. Colour proportional to scaled log-transformed expression. For human ovarian supporting cells only, 'o' denotes TF whose binding motifs are differentially accessible (that is, TF can bind their potential targets); 'a' denotes TF whose targets are also differentially expressed (that is, differentially activated TF) and asterisk denotes TF that meets both 'o' and 'a' conditions. Conservation heatmap (right) highlights significant overexpression (log_2_ fold change > 0 and FDR < 0.05) in each species. TFs whose upregulation is conserved across species are highlighted with bold/coloured labels. **d**, Dot plots showing scaled *z* scored expression of genes coding for interacting ligand–receptor proteins (CellPhoneDB) in supporting and germ cell states in the outer cortex, inner cortex and primordial follicles. Specific interacting partners are linked with a matching symbol. CoelEpi, coelomic epithelium; Expr, expressed; FGC, fetal germ cells; preGC, pregranulosa cells; granulosa, developing granulosa.

In mouse ovaries, the coelomic epithelium differentiates into the ovarian surface epithelium and initiates a second wave of cortical pregranulosa cells, independent of RSPO1/WNT4–β-catenin signalling^5,21^. In humans, we also define a second wave of granulosa cells (preGC-IIa/b) appearing after 8 PCW (Fig. 2d), downregulating *RSPO1/WNT4* (Fig. 4a) and forming a gradient from the outer (preGC-IIa) to the inner cortex (preGC-IIb; Fig. 4b and Supplementary Note 6). PreGC-IIa coappear in space (outer cortex) and time (mid-8 PCW) with OSE (*UPK3B*^+^, *LHX2*^+^, *IRX3*^+^), and express the retinoic acid inhibitor *CYP26B1* (meiosis inhibitor) as well as low amounts of *FOXL2*. PreGC-IIb appear at 11 PCW, and upregulate *FOXL2* and *BMP2*. At around 17 PCW, developing granulosa cells expressing folliculogenesis markers (*NOTCH3*^*+*^, *HEYL*^*+*^) and retinol dehydrogenase (*RDH10*^*+*^) appear in the inner cortex. The first wave of pregranulosa (preGC-I) is restricted to the medulla as the ovary develops.

**Extended Data Fig. 8:**
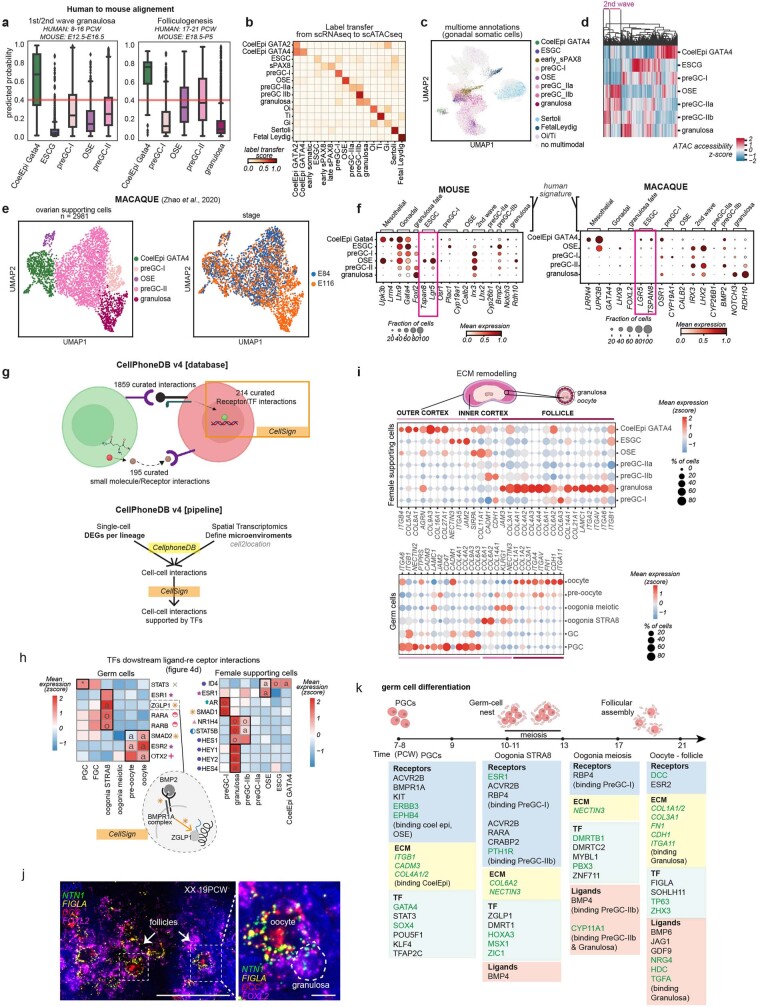
Second wave of fetal pre-granulosa. **a**, Boxplots of the predicted probabilities (Y-axis) of the label transfer from human to mouse supporting cells (X-axis) in the ovaries around the time of the second wave of pre-granulosa cells (8-16 post-conceptional weeks (PCW) human, embryonic day (E) 12.5-E16.5 mouse, n = 10,042 cells; left) and around the time of folliculogenesis (17-21 PCW human, E18.5- postnatal day (P)5 mouse, n = 5,296 cells; right). The box extends from the lower to upper quartile values of the data, with a line at the median. The whiskers extend from the box to show the range of the data. Flyer points are those past the end of the whiskers. **b**, Heatmap reporting label transfer scores from human scRNA-seq to scATAC-seq somatic cell data of matched individuals. **c**, UMAP (uniform manifold approximation and projection) of somatic cells in the human scATAC-seq dataset labelled by the cell state identified in snRNA-seq data from the cell-coupled snRNA-seq/snATAC-seq profiling. **d**, Hierarchical clustering of z-scores for each cis-co-accessibility network (CCAN) identified in human ovarian supporting cells in the human scATAC-seq dataset. **e**, UMAP projections of somatic cells in the macaque scRNA-seq dataset re-analysed from Zhao *et al*., 2020 labelled by cell type and stage. **f**, Dot plots showing the variance-scaled, log-transformed expression of genes (X-axis) characteristic of ovarian supporting cells (Y-axis) in mouse (left) and macaque (right) scRNA-seq data. Top-layer groups marker genes by categories. **g**, (top) Diagram showing the information added in the updated version of CellPhoneDB database (CellPhoneDB v4), which includes: (i) 534 novel (1,852 total) ligand-receptor interactions; (ii) 194 novel interactions mediated by small molecules; (iii) 186 novel curated links between ligand-receptor and transcription factors (CellSign module). (bottom) Diagram showing the new statistical framework to infer active cell-cell interaction partners. It includes an additional step to indicate active ligand-receptor partners in our data based on the activation of downstream signals on the receiver cell (CellSign module). Downstream signals are calculated based on TF expression and TF activity from scRNA-seq and scATAC-seq data. **h**, Heatmap showing the expression of TF downstream the receptors (CellSign) upregulated in germ and supporting cells (shown in Fig. 4d). Colour proportional to scaled log-transformed expression. Symbols highlight TF status, as in (Fig. 4b). Specificity between receptors and the corresponding downstream TF are indicated with a symbol matching the upstream receptors in Fig. 4d. **i**, Dot plots showing scaled log- transformed expression of genes coding for interacting extra-cellular matrix (ECM) proteins in supporting (top) and germ (bottom) cells states. **j**, High-resolution imaging of representative gonadal section of a human fetal ovary (19PCW), with intensity proportional to smFISH signal for *NTN1* (green, granulosa), *FIGLA* (yellow, oocytes), *DCC* (red, oocyte), *FOXL2* (magenta, granulosa); n = 2. White dashed rectangles highlight follicles and the enlarged gonadal region. Scale bars = 100 µm and 10 µm in the magnified region. **k**, Schematic illustration of main TFs, receptors, ligands and extracellular molecules regulating germ cell differentiation influenced by the granulosa lineage. New molecules identified in our study are highlighted in green. CoelEpi = coelomic epithelium; E = embryonic day; ESGC = early supporting gonadal cells; FGC = fetal germ cells; Gi = gonadal interstitial; Oi = ovarian interstitial; OSE = ovarian surface epithelium; P = postnatal day; PCW = post-conceptional week; PGC = primordial germ cells; preGC = pre-granulosa cells; TF = transcription factor; Ti = testicular interstitial; sPAX8 = supporting PAX8. Source data

Despite the spatiotemporal similarities between preGCs across species, projection of the human supporting signatures onto the mouse counterpart using an SVM classifier shows divergent transcriptomic programmes (median prediction probability <0.4; Extended Data Fig. 8a). We combined transcriptomics with chromatin accessibility to identify the TF that regulates the granulosa waves in humans (Extended Data Fig. 8b–d). Accordingly, we find TF modules are well preserved between humans and macaques but show essential differences in mice (Fig. 4c, Extended Data Fig. 8e,f and Supplementary Table 7). OSE activates the primate-specific TF *LHX2*, which is kept active in preGC-IIa (Fig. 4c). As they differentiate, preGC-IIb cells upregulate *FOXL2* and express WNT-induced TFs (*HIF1A*^*+*^, *FOXO1*^*+*^, *FOXP1*^*+*^), a programme shared by medullary preGC-Is, suggesting there is a higher WNT environment deeper in the ovary. Developing granulosa cells in primates upregulate the steroid hormone receptor *NR1H4* and the developmental factor *PBX3*.

To study how human pregranulosa cells in the distinct cortical and medullary microenvironments could influence germ cell differentiation, we expanded our CellPhoneDB database to (1) include non-peptide ligands and (2) link receptors with their downstream TFs (CellSign module) (Extended Data Fig. 8g, Supplementary Table 8 and Supplementary Note 6). PreGC-IIa cells, present in the outer ovarian cortex, express chemoattractants (for example, *NRG1*) and survival factors (for example, *KITLG*), with STAT3 downstream of KIT active in PGCs (Fig. 4d and Extended Data Fig. 8h). PreGC-IIb cells, located in the inner cortex, express ligands involved in meiosis initiation (for example, retinoic acid by *ALDH1A1*) and oogenesis (for example, *BMP2*) to support PGC differentiation. In the medulla, preGC-Is upregulate enzymes involved in oestrogen production (*HSD17B6* and *CYP19A1*). At roughly 17 PCW, preGC-IIb cells differentiate into developing granulosa cells, which surround the oocyte to mediate follicle formation and/or regulate oocyte survival. We uncover a unique composition of extracellular matrix proteins in follicles (Extended Data Fig. 8i), as well as new granulosa-to-oocyte interaction candidates for mediating successful follicular assembly (Fig. 4d). An example is netrin-1 (*NTN1*) and its receptor *DCC*, which are involved in axon guidance, cell migration and apoptosis (Extended Data Fig. 8j,k).

## Two testis-specific resident macrophages

**Fig. 5:**
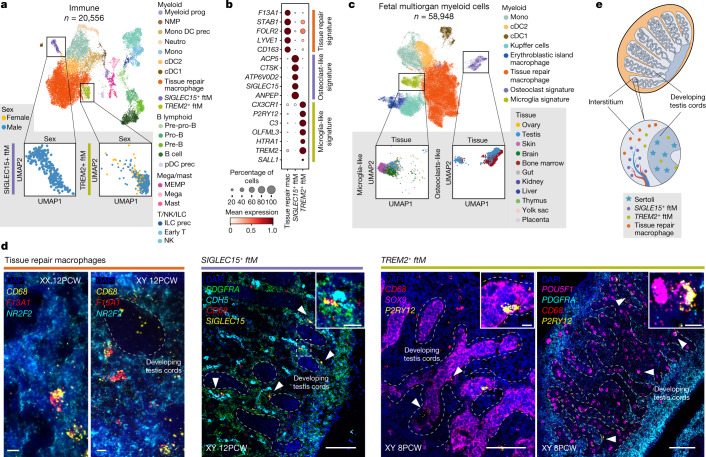
Tissue-resident macrophages in the developing testes. **a**, UMAP of immune cell states (colour) in the human scRNA-seq data (*n* = 20,556). Doublets and low-quality control cells were removed. Eleven samples were enriched for immune (CD45^+^) cells. Zoomed-in UMAPs show *SIGLEC15*^*+*^ and *TREM2*^*+*^ fetal testicular macrophages (ftMs) labelled by sex. **b**, Dot plot showing variance-scaled, log-transformed expression of marker genes (*y*-axis) for the identified macrophage subsets (*x*-axis). **c**, UMAP projections of integrated myeloid cells (colour) from several embryonic/fetal tissues (*n* = 58,948). Zoomed-in UMAPs show osteoclast and microglia signature macrophages labelled by tissue of origin. **d**, High-resolution imaging of representative human gonadal sections with intensity proportional to smFISH signal for RNA markers. Left, 12 PCW testis and ovary stained for *CD68* (yellow, macrophages), *F13A1* (red, tissue-repair macrophages) and *NR2F2* (cyan, mesenchymal) (*n* = 2). Middle, 12 PCW testis stained for *PDGFRA* (green, mesenchymal), *CDH5* (cyan, endothelial), *CD68* (red, macrophages) and *SIGLEC15* (yellow, *SIGLEC15*^+^ ftMs). *SIGLEC15*^+^ ftMs (white arrows) are outside the testis cords in proximity to endothelial cells (*n* = 5). Right, 8 PCW testis stained for *SOX9* (magenta, Sertoli (*n* = 5)), *POU5F1* (magenta, PGCs (*n* = 2)), *CD68* (red, macrophages), *P2RY12* (yellow, *TREM2*^*+*^ ftMs) and *PDGFRA* (cyan, mesenchymal). *TREM2*^*+*^ ftMs (white arrows) are adjacent to the germ and Sertoli cells. White dashed rectangles highlight gonadal regions magnified; scale bars, 100 and 10 µm in magnified regions; testicular developing cords are delineated with dashed lines. **e**, Schematics illustrating the spatial location of the distinct testicular macrophage populations. cDC, conventional dendritic cells; ftM, fetal testicular macrophages; ILC, innate lymphoid cells; mega, megakaryocytes; MEMP, megakaryocyte-erythroid-mast cell progenitors; mono, monocytes; neutro, neutrophils; NMP, neutrophil-myeloid progenitors; NK, natural killer cells; pDC, plasmacytoid dendritic cell; prec, precursor; Pre-B, pre-B cells; Pre-pro-B, pre-pro-B cells; Pro-B, pro-B cells; prog, progenitor; T, T cells.

**Extended Data Fig. 9:**
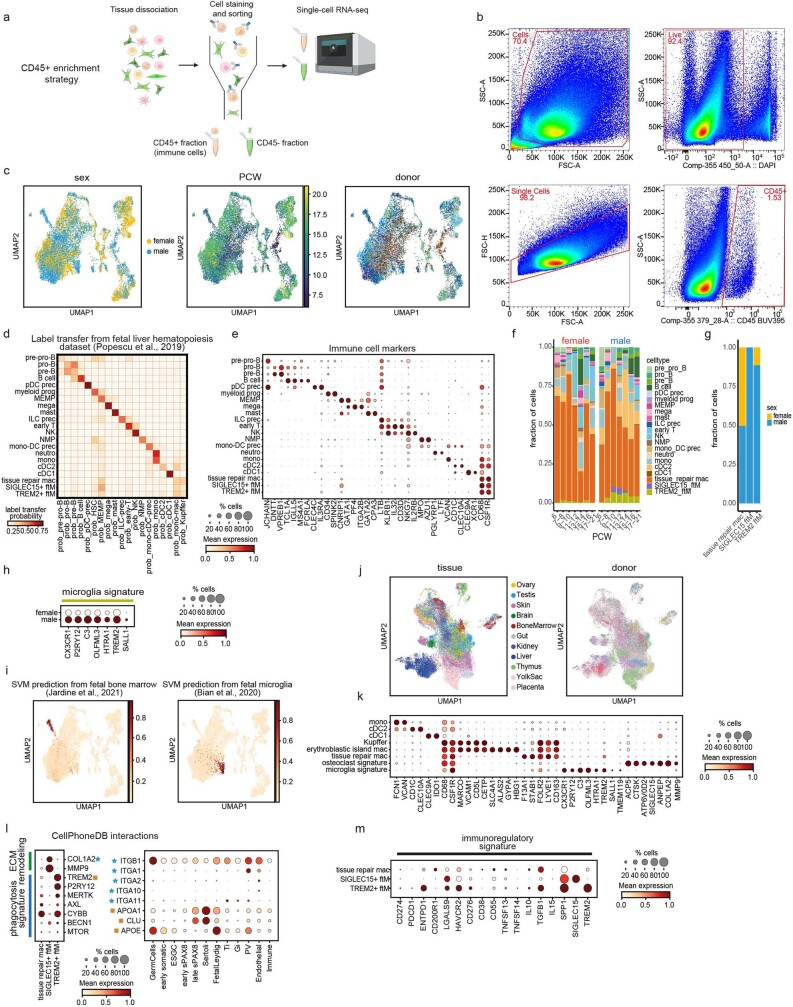
Tissue-resident macrophages in the developing testes. **a**, Schematics illustrating the CD45+ enrichment strategy for gonadal and extragonadal samples. The 11 samples that were sorted with the pan-leukocyte marker CD45 are from the following developmental stages: 6, 11, 12 PCW males, and 7.5, 8.4, late 8, 9, 11, 11, 14, 17 PCW females. **b**, Gating strategy to sort immune cells in gonadal samples for a representative donor (F93). Cells were gated on live, singlets and CD45+. **c**, UMAP projections of immune cells labelled by sex, PCW and donor. **d**, Heatmap showing label transfer scores from the fetal liver hematopoiesis dataset (Popescu *et al*., 2019) to our gonadal immune dataset using a Support Vector Machine (SVM) classifier. Low probabilities assigned to neutrophils, which were not defined in the liver dataset, and to macrophages **e**, Dot plot showing variance-scaled, log-transformed expression of marker genes expressed in the identified immune subsets. **f**, Barplot showcasing the proportions of immune cells labelled by cell state and classified by sex and developmental stage. **g**, Barplot showcasing the proportion of cells belonging to each identified macrophage population in females and males. **h**, Dot plot showing the variance-scaled, log-transformed expression of microglia markers in the cluster of *TREM2+* ftM in both sexes reveals that the few female cells that belong to this cluster do not express the key markers. **i**, Predicted probability of bone marrow osteoclasts from Jardine *et al*., 2021 (left) and brain microglia cells from Bian *et al*., 2020 (right) onto our gonadal immune manifold using a SVM classifier. **j**, UMAP (uniform manifold approximation and projection) of the multi-organ integrated fetal myeloid dataset labelled by tissue and donor. **k**, Dot plot showing variance-scaled, log-transformed expression of marker genes expressed in the identified cell populations from the multi-organ integrated fetal myeloid dataset. **l**, Dot plots showing variance-scaled, log-transformed expression of interacting ligands and receptors in the *SIGLEC15+* and *TREM2+* ftM and gonadal cell populations. Interacting partners (CellPhoneDB) are indicated with a matching symbol. **m**, Dot plot showing variance-scaled, log-transformed expression of immunoregulatory markers in human gonadal macrophages. cDC = conventional Dendritic cells; ECM = extracellular matrix; ESGC = early supporting gonadal cells; ftM = fetal testicular macrophages; Gi = gonadal interstitial; ILC prec = innate lymphoid cell precursors; Mac = macrophages; Mast = mast cells; Mega = megakaryocytes; MEMP = megakaryocyte-erythroid-mast cell progenitors; Mono = monocytes; Neutro = neutrophil; NMP = neutrophil-myeloid progenitors; NK = Natural Killer cells; pDC = plasmacytoid Dendritic cell; PV = perivascular; PCW = post-conceptional week; Prec = precursor; Pre_B = pre-B cells; Pre_pro_B = pre-pro-B cells; Pro_B = pro-B cells; Prob_ = probability; sPAX8 = supporting PAX8; Gi = gonadal interstitial; T = T cells; Ti = testicular interstitial.

**Extended Data Fig. 10:**
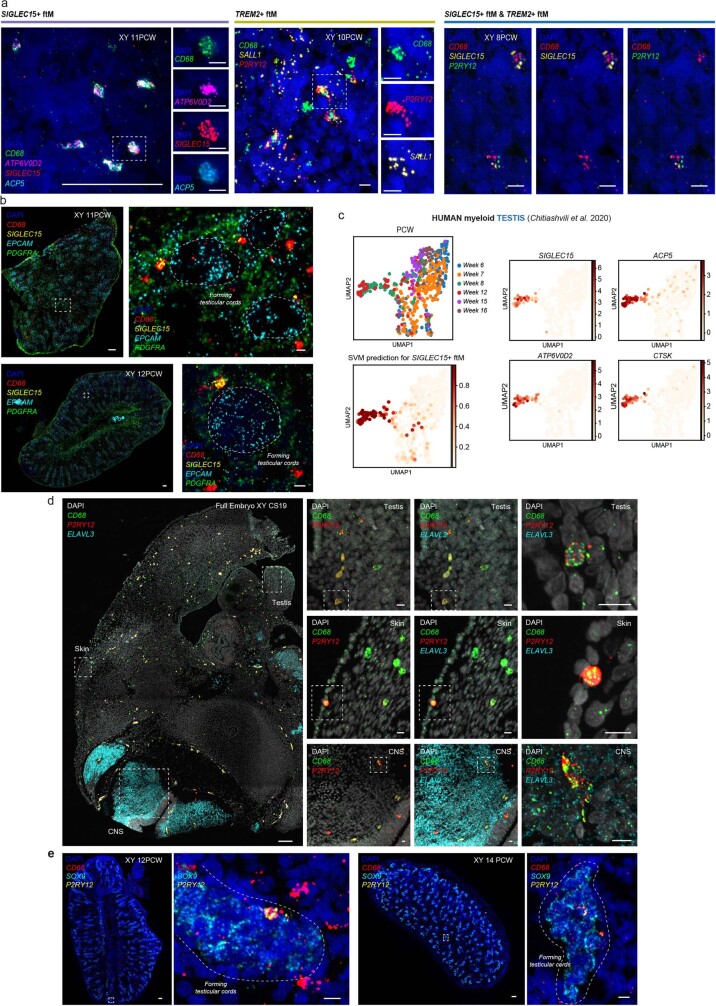
Macrophages smFISH panels. **a**, High-resolution imaging of representative sections of a fetal testes, with intensity proportional to smFISH signal for RNA markers. (left) 11 PCW testis stained for *CD68* (green, macrophages), *SIGLEC15* (red, *SIGLEC15+* ftM), *ATP6V0D2* (magenta, *SIGLEC15+* ftM), *ACP5* (cyan, *SIGLEC15+* ftM); n = 5. (middle) 10 PCW testis stained for *CD68* (green, macrophages), *P2RY12* (red, *TREM2+* ftM), *SALL1* (yellow, *TREM2+* ftM); n = 4. (right) 8 PCW testis stained for *CD68* (red, macrophages), *SIGLEC15* (yellow, *SIGLEC15+* ftM) and *P2RY12* (green, *TREM2+* ftM); n = 3. **b**, High-resolution imaging of representative sections of two fetal testes (11 and 12 PCW), with intensity proportional to smFISH signal for *EPCAM* (cyan, high = epithelial cells; low = Sertoli and germ cells), *PDGFRA* (green, mesenchymal cells), *CD68* (red, macrophages), *SIGLEC15* (yellow, *SIGLEC15+* ftM); n = 7. **c**, (top left) UMAP (uniform manifold approximation and projection) of myeloid cells from Guo et al., 2021 labelled by PCW. (bottom left) Predicted probability of *SIGLEC15+* ftM from our data onto myeloid cells from Guo et al., 2021 using a Support Vector Machine classifier. (right) UMAP projections of myeloid cells from Guo et al., 2021 showing the expression of *SIGLEC15+* ftM marker genes. **d**, High-resolution large-area imaging of a representative section of a full male embryo (Carnegie Stage CS19), with intensity proportional to smFISH signal for *CD68* (green, macrophages), *P2RY12* (red, *TREM2+* ftM and microglia), *ELAVL3* (cyan, neural cells). White dashed rectangles highlight the magnified regions from the following organs: testis (top), skin (middle), spinal cord (labelled as CNS = central nervous system) (bottom); n = 1 **e**, High-resolution imaging of representative gonadal sections of two fetal testes (12 and 14 PCW), with intensity proportional to smFISH signal to *SOX9* (cyan, Sertoli cells), *CD68* (red, macrophages), *P2RY12* (yellow, *TREM2+* ftM); n = 5. ftM = fetal testicular macrophages; PCW = post-conceptional week; prob_ = probability. For all smFISH panels, unless otherwise specified, white dashed rectangles highlight gonadal regions magnified; scale bars = 100 µm and 10 µm in magnified regions; developing testis cords are delineated with dashed lines.

Tissue-resident macrophages have a role in mouse testicular development and function^22,23^. To comprehensively characterize them in humans, we sorted cells from 11 samples using the pan-leukocyte marker CD45 and integrated them with immune cells from the main analyses (Fig. 5a, Extended Data Fig. 9a–f, Supplementary Table 1 and Supplementary Note 7). We defined two testis-specific macrophage populations using scRNA-seq and validated them with smFISH: (1) *SIGLEC15*^*+*^ fetal testicular macrophages (ftMs), with an osteoclast-like signature (*SIGLEC15*, *ACP5*, *ATP6V0D2*; refs. ^24–27^) and (2) *TREM2*^*+*^ ftMs, with a microglia-like signature (*TREM2, P2RY12*, *SALL1*; refs. ^28–30^) (Fig. 5a,b, Extended Data Fig. 9g,h, Extended Data Fig. 10a and Supplementary Table 9). *SIGLEC15*^*+*^ and *TREM2*^*+*^ ftMs are rare populations in comparison to the tissue-repair macrophages characteristic of all developing tissues (2.8% *SIGLEC15*^*+*^ ftMs, 5% *TREM2+* ftMs, 92.2% tissue-repair macrophages). Integration and projection using SVM of scRNA-seq datasets of myeloid cells in other developing organs^28,31–35^ onto our gonadal immune manifold validated the shared transcriptomics profile between *SIGLEC15*^*+*^ ftMs and osteoclasts, and between *TREM2*^*+*^ ftMs and microglia (Fig. 5c and Extended Data Fig. 9i–k).

With the aid of structural gonadal markers, smFISH imaging located tissue-repair macrophages (*CD68*^*+*^, *F13A1*^+^) and *SIGLEC15*^+^ ftMs (*CD68*^*+*^, *SIGLEC15*^*+*^) in the interstitial space (*PDGFRA*^*+*^ or *NR2F2*^*+*^) (Fig. 5d and Extended Data Fig. 10b). *SIGLEC15*^+^ ftMs are close to endothelial cells (*CDH5*^*+*^) in the testes (Fig. 5d) and express *COL1A2*, which can potentially interact with the integrins (α1/β1, α2/β1, α10/β1 and α11/β1) expressed by endothelial and mesenchymal cells (Extended Data Fig. 9l). *SIGLEC15*^+^ ftMs also express the remodelling molecule *MMP9* and their numbers decrease in later stages of development (Extended Data Fig. 10c), suggesting a role in promoting mesonephric endothelial cell migration^36^, a transient process required for testis cord formation (roughly 8–14 PCW). In addition, *SIGLEC15*^*+*^ ftMs express *LGALS9* and *SPP1,* in keeping with a potential immunoregulatory role for this cell type (Extended Data Fig. 9m).

*TREM2*^*+*^ ftMs are often found inside the testis cords (Fig. 5d and Extended Data Fig. 10d,e), where they are predicted to communicate with Sertoli and germ cells by the interaction between *TREM2* and apolipoproteins (*CLU*, *APOA1*, *APOE*) (Extended Data Fig. 9l). *TREM2*^*+*^ ftMs also have their phagocytosis machinery active (*MERTK*, *AXL*, *CYBB*, *BECN1*, *MTOR*) (Extended Data Fig. 9l) and express immunomodulatory molecules (*HAVCR2*, *ENTPD1*, *CD276*, *IL10*, *TREM2*) (Extended Data Fig. 9m). This result indicates a role of *TREM2*^*+*^ ftMs in removing damaged or apoptotic cells while minimizing inflammation and oxidative stress that could damage maturing germ cells^37^ (Fig. 5e and Supplementary Note 7).

## Discussion

We generated a harmonized atlas of human and mouse gonadal development to identify new gonadal somatic cell types and their underlying regulatory mechanisms. First, we describe ESGCs, a bipotent transient population whose numbers peak at the time of sex determination and that connects the coelomic epithelium with Sertoli cells and the first wave of pregranulosa cells. Accordingly, ESGCs are the first cells to express the testis-determining factor *SRY* in XY gonads and, in humans, express stem-cell markers such as *TSPAN8* and *LGR5*. For the first time, to our knowledge, these markers uniquely expressed by the bipotent supporting progenitor population are defined in humans. Previously, WT1 and NR5A1 were used to identify an equivalent population in mice^2^, but we show that these markers are broadly expressed by other gonadal somatic cells. Second, around the onset of sex determination, we define a previously uncharacterized gonadal supporting-like population located at the gonadal–mesonephric border, which we term sPAX8s. In humans, after 9 PCW, sPAX8s remain at the poles of the developing cords in males, where the rete testis develops, but are virtually absent in females. sPAX8s express canonical markers of the supporting lineage and it is likely that their unique functions were previously attributed to the other supporting cells (that is, granulosa or Sertoli cells). Third, we identify a first wave of medullary and a second wave of cortical pregranulosa cells in humans, similar to mice^5,21,38^. Using a revised version of CellPhoneDB, we show that the spatial microenvironments defined by the distinct pregranulosa cell subsets in human ovaries regulate germ cell development. Despite the similar spatiotemporal patterns in humans and mice, we show that certain regulatory programmes differ; for example, *LGR5*, characteristic of second-wave pregranulosa cells in mice^5,38^, is restricted to ESGCs in humans. *LGR5* thus marks different populations in mice and humans, highlighting the need for human–mouse harmonized atlases. Fourth, we identify *SIGLEC15*^+^ and *TREM2*^*+*^ ftMs with an osteoclast- and microglia-like profile, respectively. *SIGLEC15*^+^ ftMs are found in the peritubular spaces surrounding the testis cords, which might aid with mesonephric endothelial cell migration^36^. *TREM2*^*+*^ ftMs are mainly located inside the testis cords, where they could help to maintain the immunoregulatory environment previously described in prepubertal testes^39,40^.

Overall, our comprehensive cellular map of human and mouse gonadal development provides a unique resource to study gonadal function, relevant to understanding infertility, differences in sex development^41^ and gonadal pathologies^42^. We foresee that the discovery of new cell populations, together with our cross-species TF alignment, will serve as a blueprint for the design of systems to differentiate gonadal somatic cells in vitro, which will affect the development of new in vitro gametogenesis protocols^43–46^.

## Methods

### Patient samples

All tissue samples used for this study were obtained with written informed consent from all participants in accordance with the guidelines in The Declaration of Helsinki 2000.

Human embryo and fetal samples were obtained from the MRC and Wellcome-financed Human Developmental Biology Resource (HDBR, http://www.hdbr.org), with appropriate maternal written consent and approval from the Fulham Research Ethics Committee (REC reference no. 18/LO/0822) and Newcastle and North Tyneside 1 Research Ethics Committee (REC reference no. 18/NE/0290). The HDBR is regulated by the UK Human Tissue Authority (www.hta.gov.uk) and operates in accordance with the relevant Human Tissue Authority Codes of Practice.

### Assignment of developmental stage

Embryos up to 8 PCW were staged using the Carnegie staging method^47^. At stages beyond 8 PCW, age was estimated from measurements of foot length and heel-to-knee length and compared with the standard growth chart^48^. A piece of skin, or if this was not possible, chorionic villi tissue, was collected from every sample for quantitative PCR analysis using markers for the sex chromosomes and autosomes 13, 15, 16, 18, 21 and 22, which are the most commonly seen chromosomal abnormalities. All samples were karyotypically normal.

### Tissue processing

All tissues for sequencing and spatial work were collected in HypoThermosol biopreservation medium and stored at 4 °C until processing. Tissue dissociation was conducted within 24 h of tissue retrieval with the exception of tissues that were cryopreserved and stored at −80 °C (Supplementary Table 1).

We used the previous protocol optimized for gonadal dissociation^8^ and this is available at protocols.io (ref. ^49^). In short, tissues were cut into <1 mm^3^ segments before being digested with Trypsin/EDTA 0.25% for 5–15 min at 37 °C with intermittent shaking. Samples less than 17 PCW were also digested using a combination of collagenase and Trypsin/EDTA, a protocol adapted from Wagner et al.^50,51^. In short, samples were first digested with collagenase 1A (1 mg ml^−1^) and liberase TM (50 µg ml^−1^) for 45 min at 37 °C with intermittent shaking. The cell solution was further digested with Trypsin/EDTA 0.25% for 10 min at 37 °C with intermittent shaking. In both protocols, digested tissue was passed through a 100 µm filter and cells collected by centrifugation (500*g* for 5 min at 4 °C). Cells were washed with PBS before cell counting.

### Cell sorting

Dissociated cells were incubated at 4 °C with 2.5 μl of antibodies in 1% FBS in Dulbecco’s PBS without calcium and magnesium (Thermo Fisher Scientific, 14190136). To isolate CD45^+^ and CD45^−^ cells, we used the antibody CD45-BUV395 BD Bioscience 563791 Clone HI30 (RUO) Flow cytometry (dilution 2.5 μl:100 μl). 4,6-Diamidino-2-phenylindole (DAPI) was used for live versus dead discrimination. Cells were sorted using a Becton Dickinson (BD) FACS Aria Fusion with five excitation lasers (355, 405, 488, 561 and 635 nm red), and 18 fluorescent detectors, plus forward and side scatter. The sorter was controlled using BD FACS DIVA software (v.7), and FlowJo v.10.3 was used for analysis.

### Single-nuclei suspension

Single-nuclei suspensions were isolated from dissociated cells when performing scATAC-seq, following the manufacturers’ instructions, and from frozen tissue sections when performing multiomic snRNA-seq/scATAC-seq. For the latter, thick (300 µm) sections were cryosectioned and kept in a tube on dry ice until subsequent processing. Nuclei were released by Dounce homogenization as described in detail in the protocols.io (ref. ^52^).

### Tissue cryopreservation

Fresh tissue was cut into <1 mm^3^ segments before being resuspended with 1 ml of ice-cold Cryostor solution (CS10) (C2874-Sigma). The tissue was frozen at −80 °C by decreasing the temperature at about 1 °C per minute. The detailed protocol is available at https://www.protocols.io/view/tissue-freezing-in-cryostor-solution-processing-bgsnjwde.

### Tissue freezing

Fresh tissue samples of human developing gonads were embedded in cold optimal cutting temperature compound (OCT) medium and flash frozen using a dry ice-isopentane slurry. The protocol is available at protocols.io (ref. ^53^).

### Tissue collection from mouse embryos

Developing ovaries, testes and mesonephros were collected from E10.5, E11.5 and E12.5 mouse embryos carrying the Oct4ΔPE-GFP transgene. Mice were housed in specific pathogen-free conditions at the UK Home Office-approved facility at the University of Cambridge. Mice were maintained with a 12 h light/12 h dark cycle, with temperature ranging from 20–24 °C and humidity of 45–65%. Embryos were genotyped to identify the gender. We included six males and three females at E10.5, six males and two females at E11.5, and three males and three females at E12.5. Sample size was not estimated. Developing gonads were dissected from the mesonephros and both organs were separately dissociated with 0.25% Trypsin/EDTA into single-cell suspensions as described for the human tissue. Tissues (gonads or mesonephros) from the same sex and stage were sequenced together. For smFISH imaging, we collected another E13.5 female embryo. For sectioning, tissues were fixed in 4% (w/v) formaldehyde solution for 2 h at 4 °C. Samples were washed with PBS and afterwards sequentially incubated with 10 and 20% (w/v) sucrose at 4 °C. After, samples were embedded in OCT and subsequently flash frozen using a dry ice-isopentane slurry. All experimental procedures were in agreement with the project licence PE596D1FE issued by the Animal Welfare Ethical Review Board committee under the UK Home Office and carried out in a Home Office designated facility, in accordance with ethical guidelines and with the UK Animals (Scientific Procedures) Act of 1986.

### Haematoxylin and eosin staining and imaging

Fresh frozen sections were removed from −80 °C storage and air dried before being fixed in 10% neutral buffered formalin for 5 min. After being rinsed with deionized water, slides were dipped in Mayer’s haematoxylin solution for 90 s. Slides were completely rinsed in 4–5 washes of deionized water, which also served to blue the haematoxylin. Aqueous eosin (1%) was manually applied onto sections with a pipette and rinsed with deionized water after 1–3 s. Slides were dehydrated through an ethanol series (70, 70, 100, 100%) and cleared twice in 100% xylene. Slides were coverslipped and allowed to air dry before being imaged on a Hamamatsu NanoZoomer 2.0HT digital slide scanner.

### Multiplexed smFISH and high-resolution imaging

Large tissue section staining and fluorescent imaging were conducted largely as described previously^54^. Sections were cut from fresh frozen or fixed frozen samples embedded in OCT at a thickness of 10 μm using a cryostat, placed onto SuperFrost Plus slides (VWR) and stored at −80 °C until stained. For formalin-fixed paraffin-embedded samples, sections were cut at a thickness of 5 μm using a microtome, placed onto SuperFrost Plus slides (VWR) and left at 37 °C overnight to dry and ensure adhesion. Tissue sections were then processed using a Leica BOND RX to automate staining with the RNAscope Multiplex Fluorescent Reagent Kit v2 Assay (Advanced Cell Diagnostics, Bio-Techne), according to the manufacturers’ instructions. Probes are listed in Supplementary Table 10. Before staining, human fresh frozen sections were post-fixed in 4% paraformaldehyde in PBS for 15 min at 4 °C, then dehydrated through a series of 50, 70, 100 and 100% ethanol, for 5 min each. Following manual pretreatment, automated processing included epitope retrieval by protease digestion with Protease IV for 30 min before probe hybridization. Mouse fixed frozen sections were subjected to the same manual pretreatment described above. Subsequently, the automated processing for these sections included heat-induced epitope retrieval at 95 °C for 5 min in buffer ER2 and digestion with Protease III for 15 min before probe hybridization. On this treatment, no endogenous fluorescence from the Oct4ΔPE-GFP transgene was observed. For formalin-fixed paraffin-embedded sections, automated processing included baking at 60 °C for 30 min and dewaxing, as well as heat-induced epitope retrieval at 95 °C for 15 min in buffer ER2 and digestion with Protease III for 15 min before probe hybridization. Tyramide signal amplification with Opal 520, Opal 570 and Opal 650 (Akoya Biosciences) and TSA-biotin (TSA Plus Biotin Kit, Perkin Elmer) and streptavidin-conjugated Atto 425 (Sigma Aldrich) was used to develop RNAscope probe channels.

Stained sections were imaged with a Perkin Elmer Opera Phenix High-Content Screening System, in confocal mode with 1 μm z-step size, using a ×20 (numerical aperture (NA) 0.16, 0.299 μm per pixel), ×40 (NA 1.1, 0.149 μm per pixel) or ×63 (NA 1.15, 0.091 μm per pixel) water-immersion objectives. Channels were as follows: DAPI (excitation 375 nm, emission 435–480 nm), Atto 425 (excitation 425 nm, emission 463–501 nm), Opal 520 (excitation 488 nm, emission 500–550 nm), Opal 570 (excitation 561 nm, emission 570–630 nm) and Opal 650 (excitation 640 nm, emission 650–760 nm).

### Image stitching

Confocal image stacks were stitched as two-dimensional maximum intensity projections using proprietary Acapella scripts provided by Perkin Elmer.

### 10X Genomics Chromium GEX (gene expression) library preparation and sequencing

For the scRNA-seq experiments, cells were loaded according to the manufacturer’s protocol for the Chromium Single Cell 5′ Kit v.1.0, v.1.1 and v.2 (10X Genomics) to attain between 2,000 and 10,000 cells per reaction. Library preparation was carried out according to the manufacturer’s protocol. Libraries were sequenced, aiming at a minimum coverage of 20,000 raw reads per cell, on the Illumina HiSeq4000 or Novaseq 6000 systems using the sequencing format: read 1, 26 cycles; i7 index, 8 cycles, i5 index, 0 cycles; read 2, 98 cycles.

For the scATAC-seq and multimodal snRNA-seq/scATAC-seq experiments, cells were loaded according to the manufacturer’s protocol for the Chromium Single Cell ATAC v.1.0 and Chromium Single Cell Multiome ATAC + Gene Expression v.1.0 to attain between 2,000 and 10,000 cells per well. Library preparation was carried out according to the manufacturer’s protocol. Libraries for scATAC-seq were sequenced on Illumina NovaSeq 6000, aiming at a minimum coverage of 10,000 fragments per cell, with the following sequencing format; read 1, 50 cycles; i7 index, 8 cycles, i5 index, 16 cycles; read 2, 50 cycles.

### 10X Genomics Visium library preparation and sequencing

Cryosections of 10 μm were cut and placed on Visium slides. These were processed according to the manufacturer’s instructions. In brief, sections were fixed with cold methanol, stained with H&E and imaged on a Hamamatsu NanoZoomer S60 before permeabilization, reverse transcription and complementary DNA synthesis using a template-switching protocol. Second-strand cDNA was liberated from the slide and single-indexed libraries prepared using a 10X Genomics PCR-based protocol. Libraries were sequenced (one per lane on a HiSeq4000), aiming for 300 million raw reads per sample, with the following sequencing format; read 1, 28 cycles, i7 index, 8 cycles, i5 index, 0 cycles and read 2, 91 cycles.

### Alignment and quantification of sc or snRNA-seq data

For each sequenced scRNA-seq library, we performed read alignment to the 10X Genomics’ GRCh38 v.3.1.0 (human) or Mm10-2020 (mouse) reference genomes, quantification and initial quality control using the Cell Ranger Software (v.3.1, 10X Genomics) using default parameters. For each sequenced multimodal snRNA-seq library, we performed read alignment to the 10X Genomics’ GRCh38 v.3.1.0 (human) reference genome, quantification and initial quality control using the Cell Ranger ARC Software (v.1.0.1, 10X Genomics) using default parameters. Cell Ranger filtered count matrices were used for downstream analysis.

### Downstream scRNA-seq analysis

#### Doublet detection

We used Scrublet for cell doublet calling on a per-library basis. We used a two-step diffusion doublet identification followed by Bonferroni-false discovery rate (FDR) correction and a significance threshold of 0.01, as described^31^. Predicted doublets were not excluded from the initial analysis, but used afterwards to flag clusters with high doublet scores.

#### Quality filters, alignment of data across different batches and clustering

For scRNA-seq libraries, we integrated the filtered count matrices from Cell Ranger and analysed them with Scanpy v.1.7.0, with the pipeline following their recommended standard practices. In brief, we excluded genes expressed by fewer than three cells and excluded cells expressing fewer than 500 genes (or 2,000 genes in mouse), more than 20% mitochondrial content (5% in mouse) or with both more than 10% mitochondrial content and fewer than 1,500 counts correctly mapped to the transcriptome. After converting the expression space to log(CPM/100 + 1), the object was transposed to gene space to identify cell cycling genes in a data-driven manner, as described^31,32^. After performing principal component analysis (PCA), neighbour identification and Leiden clustering, the members of the gene cluster including known cycling genes (*CDK1*, *MKI67*, *CCNB2* and *PCNA*) were flagged as the data-derived cell cycling genes and discarded in each downstream analysis. We identified highly variable genes (*n* = 2,000) using Seurat v3 flavour on the raw counts, which were used to correct for batch effect with single-cell variational inference (scVI) v.0.6.8. In the analysis of human scRNA-seq, we corrected for sample source and donor effect in both the main and the germ and somatic reanalysis. In the analysis of mouse scRNA-seq we corrected for sample effect and origin of the dataset, this last if combined with external data (below). The resulting latent representation of each cell in the dataset was used for neighbour identification, Leiden clustering and uniform manifold approximation and projection (UMAP) visualization.

General analysis was done separately on males and females in each species. Germ, gonadal somatic, endothelial and immune cells were subsequently reanalysed integrating both sexes into the same manifold, using the approach described in the previous paragraph. Furthermore, gonadal somatic cells from samples at the time of sex specification (younger than CS23) were further reanalysed for fine-grained annotation and validation.

#### Mouse gonad data

We combined our in-house mouse raw counts matrix with the raw count matrices from the ovarian samples profiled by Niu et al.^5^, comprising E11.5 to P5 developmental stages (GSE136441)^5^. For the Niu et al.^5^ dataset, we excluded cells that expressed fewer than 1,000 genes or more than 20% of mitochondrial genes.

For the analysis of mouse germ cells, we also included the mouse dataset generated by Mayère et al.^10^, which contains germ cells from mice from E10 to E18 developmental stages (GSE136220)^10^. ENSEMBL gene IDs provided by the authors were converted to gene names using the appropriate genome build (GRCm38.p5). We filtered out cells that expressed fewer than 1,000 genes or more than 20% of mitochondrial genes. Next, we concatenated male and female mouse germ cells data from our general analysis (already including data from Niu et al.^5^) with the germ cells dataset from Mayère et al.^10^, keeping the genes shared between the three datasets. The resulting matrix was integrated by sample and origin of the dataset using scVI on the basis of the procedure described above.

#### Macaque gonads data

In addition, we downloaded a macaque dataset profiling fetal ovaries at stages E84 and E116 (GSE149629)^11^ and included it in our cross-species comparison of germ and female somatic cells. Owing to low sequencing depth, we filtered out cells expressing fewer than 300 genes and more than 20% of mitochondrial genes.

As for mice, macaque gene identifiers were converted to human genes using ENSEMBL Biomart multi-species comparison filter. Genes with several mappings were discarded.

#### Annotation of scRNA-seq datasets cross-species

Identification, labelling and naming of the unbiased clusters was carried out on each species individually using a manual approach that we validated using a SVM classifier (see Cross-species comparison section below). For the manual approach, we first identified cluster-specific genes that we used to classify clusters into main cell types on the basis of bona fide marker genes previously reported in the literature. Next, we refined the annotation accounting for the spatiotemporal dynamics in each sex.

To identify marker genes specific to a cluster, we used the TF-IDF approach from the SoupX package v.1.5.0 (ref. ^55^) in R v.4.0.3. To estimate the cell cycle phase of each cell (that is, G1, S or G2/M), we aggregated the expression of G2/M and S phase markers and classified the barcodes following the method described in ref. ^56^ implemented in Scanpy score_genes_cell_cycle function. We discarded the clusters that: (1) were specific to a single donor; (2) had a higher average doublet score; (3) had lower numbers of expressed genes with no distinctive gene expressed (from TF-IDF approach) or (4) were enriched for marker genes for erythroid cells (red blood cells) and likely to be cell-free messenger RNA soup^55^.

#### Cross-species comparison

We compared the transcriptional signatures of the cell types identified in our human scRNA-seq to their mouse counterparts, considering all developmental stages combined. Mouse gene identifiers were converted to human genes using ENSEMBL Biomart multi-species comparison filter. Genes with several mappings were discarded. Furthermore, genes associated with the cell cycle were removed to avoid biases. Before training the model, human cell types were downsampled to the cell type with the lowest number of cells to obtain a balanced dataset. Here, 75% of the data were used for training the model and 25% of the data were used to test the model. Raw counts were normalized and log-transformed, and the 300 most highly variable genes were selected. We then trained an SVM classifier (sklearn.svm.SVC) on human data and projected the cell type annotations onto the mouse datasets. By doing so, we obtained a predicted probability value that each cell in the mouse and macaque dataset corresponded to every given human cell type annotation. To study the transcriptomic similarity of a given cell type across species, we compared the estimated probabilities between human–mouse matching cell types and visualized them with boxplots. A detailed description of the workflow used for cross-species comparison is reported in Supplementary Note 2.

#### Agreement with external human gonads data

We evaluated the consistency between the main lineages identified in our study with the Smart-seq2 dataset of gonadal cells from Li et al.^7^ (GSE86146). From Li et al.^7^, we downloaded the normalized transcripts per million matrix and annotated their cells using the ‘FullAnnot’ field provided in the S1 table of the publication. We used the scmap tool^57^ to project the Li et al. annotations onto our dataset, using a similarity cut-off of 0.5 to retrieve high confidence alignment, on each sex separately. To speed up computational times, we downsampled our dataset to 50% size. Li et al.’s annotations were visualized onto the male and female UMAPs, respectively.

To validate the new ESGCs population, we queried the 10X scRNA-seq dataset of developing testis from GSE143356 (ref. ^58^) analysed by Guo et al.^59^. Here, we downloaded the raw expression count matrix, and excluded cells expressing fewer than 300 genes and more than 20% of mitochondrial genes. We carried out downstream analysis as previously described for UMAP visualization. Finally, we trained a SVM classifier (sklearn.svm.SVC) on our early human male somatic cells (<CS23) and projected cell type annotations onto the somatic cells identified by Guo et al.^59^ in equivalent stages (6, 7, 8 PCW only). The label transfer workflow is analogous to that described for cross-species comparison (Supplementary Note 2), except for the initial ENSEMBL gene ID conversion, which is not necessary in this case because we are transferring labels between human datasets.

#### Analysis of immune cells in the gonads

Cell Ranger filtered count matrices of CD45^+^ enriched samples were processed using the workflow described above for the main scRNA-seq analysis (doublet detection, alignment of data across different batches with scVI and clustering). These cells were then merged with the cluster of immune cells from the non-enriched samples. The resulting clustered manifold was preliminary annotated by transferring labels from a publicly available dataset of human fetal liver haematopoiesis^31^. Developing liver scRNA-seq raw counts were downloaded from ArrayExpress (E-MTAB-7407), processed with Scanpy v.1.7.0 workflow described above for the main scRNA-seq analysis and filtered on the basis of the expression of CD45 (*PTPRC*) to exclude non-immune cells. We then trained a SVM classifier (sklearn.svm.SVC) on the filtered liver dataset and used it to predict cell types on our gonadal immune dataset. The label transfer workflow is analogous to that described for cross-species comparison (Supplementary Note 2), except for the initial ENSEMBL gene ID conversion, which is not necessary in this case as we are transferring labels between human datasets. Predicted cell type annotations were validated or disproved by looking at the expression of known marker genes.

To study the unique profile of our gonadal macrophages, we downloaded immune cells from several developing tissues: liver, skin, kidney, yolk sac, gut, thymus, placenta, bone marrow and brain^28,31–35^. Raw sequencing data were downloaded from ArrayExpress (E-MTAB-7407, E-MTAB-8901, E-MTAB-8581, E-MTAB-0701, E-MTAB-9801) or Gene Expression Omnibus (GEO) (GSE141862). For all datasets, we filtered out cells expressing fewer than 300 genes and more than 20% of mitochondrial genes. Downstream data analyses for these datasets were performed with the Scanpy v.1.7.0 workflow analogously to what is described in the main scRNA-seq analysis section above. Myeloid cells from fetal liver, skin, kidney, yolk sac, gut, thymus, placenta, bone marrow and brain datasets were selected on the basis of the expression of established myeloid markers (*CD14*, *CD68*, *CSF1R*). We then combined the resulting myeloid dataset with our gonadal myeloid cells and used scVI with a combined batch of donor and sample to integrate across the different organs.

Projection of fetal osteoclasts from Jardine et al.^35^ and microglial cells from Bian et al.^29^ onto our immune dataset was done using an SVM model. Similarly, we trained an SVM model on our gonadal macrophages and projected the cell type annotations onto fetal testicular myeloid cells from Chitiashvili et al.^58^. The label transfer workflow is analogous to that described for cross-species comparison (Supplementary Note 2), except for the initial ENSEMBL gene ID conversion, which is not necessary in this case as we are transferring labels between human datasets

#### Trajectory inference in the germ and early somatic lineages

For both germ and early somatic cells, we modelled differentiation trajectories and conducted pseudotime analysis by ordering cells along the reconstructed trajectory with Palantir (v.1.0.0)^60^ following their tutorial. In brief, cells were subsampled to balance cell type and sex contribution (*n* = 500 for germ and *n* = 150 for somatic cells). The top 2,000 highly variable genes were used for PCA. Next, we determined the diffusion maps from the PCA space (with five top components), and projected the diffusion components onto a t-SNE low dimensional embedding to visualize the data. Finally, we used the function run_palantir (with num_waypoints = 500) to estimate the pseudotime of each cell from the root cell. The barcode with the highest normalized expression of *POU5F1* (PGC marker) or *UPK3B* (mesothelial marker) was used as the cell of origin in the germ and early somatic analyses, respectively. Terminal states were determined automatically by Palantir.

For samples at the time of sex specification, we computed RNA velocities^61^ to model early somatic development with scVelo (v.0.2.4)^62^ following their tutorial. Analysis was done on each sample separately in humans and mice. First, we used STARsolo to quantify spliced and unspliced counts, keeping the same 10X Genomics genome references used in Cell Ranger before. Next, we reprocessed the somatic cells (only cells at G1 phase) from each sample independently, performed PCA on the top 2,000 highly variable genes, neighbour identification and UMAP projection to visualize previously annotated cell types. Doublets and low quality control were discarded with unbiased Leiden clustering if necessary. We also excluded extragonadal coelomic epithelium *GATA2*^+^. Using scVelo, we computed the RNA moments and estimated velocities with ‘stochastic’ mode. Next, with scVelo we combined transcriptional similarity-based trajectory inference with directional RNA velocity and generated the velocity graph on the basis of cosine similarities. To further characterize the cell fate decision process in an unbiased way, we leveraged the RNA moments with the CellRank package (v.1.5.1). Specifically, CellRank uses a random walk model to learn directed, probabilistic state-change trajectories and determine initial and terminal states. We set the number of terminal states to four, letting CellRank determine the number of initial states. We extracted the fate probability of each cell ending up in one of the terminal states.

### Alignment, quantification and quality control of ATAC data

We processed scATAC-seq libraries (read filtering, alignment, barcode counting and cell calling) with 10X Genomics Cell Ranger ATAC pipeline (v.1.2.0) using the prebuilt 10X’s GRCh38 genome (v.3.1.0) as reference. We called the peaks using an in-house implementation of the approach described in Cusanovich et al.^63^ (available at https://github.com/cellgeni/cellatac, revision 21-099). In short, the genome was broken into 5 kb windows and then each cell barcode was scored for insertions in each window, generating a binary matrix of windows by cells. Matrices from all samples were concatenated into a unified matrix, which was filtered to retain only the top 200,000 most commonly used windows per sample. Using Signac (https://satijalab.org/signac/ v.0.2.5), the binary matrix was normalized with TF-IDF followed by a dimensionality reduction step using singular value decomposition. Latent semantic indexing was clipped at ±1.5. The first latent semantic indexing component was ignored as it usually correlates with sequencing depth (technical variation) rather than a biological variation^63^. The 2–30 top remaining components were used to perform graph-based Louvain clustering. Next, peaks were called separately on each cluster using macs2 (ref. ^64^). Finally, peaks from all clusters were merged into a master peak set (that is, peaks overlapping in at least one base pair were aggregated) and used to generate a binary peak by cell matrix, indicating any reads occurring in each peak for each cell.

### Downstream scATAC-seq analysis

#### Quality filters, alignment of data across different batches and clustering

To obtain a set of high-quality peaks for downstream analysis, we filtered out peaks that (1) were included in the ENCODE blacklist, (2) had a width outside the 210–1,500 bp range and (3) were accessible in less than 4% of cells from a cellatac cluster. Low-quality cells were also removed by setting to 5.5 the minimum threshold for log_1_p transformed total counts per cell.

We adopted the cisTopic approach^65,66^ v.0.3.0 for the core of our downstream analysis. cisTopic uses latent Dirichlet allocation to estimate the probability of a region belonging to a regulatory topic (region-topic distribution) and the contribution of a topic within each cell (topic-cell distribution). The topic-cell matrix was used for constructing the neighbourhood graph, computing UMAP projections and clustering with the Leiden algorithm. Donor effects were corrected using Harmony^67^ (theta = 0). Cell doublets were identified and removed using scrublet^68^.

#### Gene activity scores

Next, we generated a denoised accessibility matrix (predictive distribution) by multiplying the topic-cell and region-topic distribution and used it to calculate gene activity scores. To integrate them with scRNA-seq data, gene activity scores were rounded and multiplied by a factor of 10^7^, as previously described^66^.

#### Cell type annotation

To annotate cell types in scATAC-seq data, we first performed label transfer from scRNA-seq data of matched individuals. We used canonical correlation analysis as a dimensionality reduction method and vst as a selection method, along with 3,000 variable features and 25 dimensions for finding anchors between the two datasets and transferring the annotations^6^. The predicted cell type annotations by label transfer were validated by importing annotations of the multiomic snRNA-seq/scATAC-seq profiling data. To visualize the correspondence between scATAC-seq final annotations and predictions from label transfer, we plotted the average label transfer score (value between 0 and 1) of each cell type in the annotated cell types in scATAC-seq data.

#### Cell type-specific *cis*-regulatory networks

Coaccessible peaks in the genome and *cis*-coaccessibility networks (CCANs) were estimated using the R package Cicero^69^ v.1.3.4.11 with default parameters. We then filtered the denoised accessibility matrix from cisTopic to keep only the peaks included in CCANs. The resulting matrix was further processed to average cells by cell type and peaks by CCAN. Finally, we z scored the matrix across CCANs and visualized the separation of CCANs by cell type by hierarchical clustering and plotting the heatmap.

### Alignment, quantification and quality control of Visium data

For each 10X Genomics Visium sequencing data, we used Space Ranger Software Suite (v.1.2.1) to align to the GRCh38 human reference genome (official Cell Ranger reference, v.2020-A) and quantify gene counts. Spots were automatically aligned to the paired H&E images by Space Ranger software. All spots under tissue detected by Space Ranger were included in downstream analysis.

### Downstream analysis of 10X Genomics Visium data

#### Location of cell types in Visium data

To spatially locate the cell states on the Visium transcriptomics slides, we used the cell2location tool v.0.05-alpha (ref. ^70^). As reference, we used scRNA-seq data from individuals of the same sex and gestational stage. We used general cell annotations from the main analysis, with the exception of the main gonadal lineages (germ, supporting and mesenchymal) for which we considered the identified subpopulations. We used default parameters with the exception of cells_per_spot that was set to 20. Each Visium section was analysed separately. Results were visualized following the cell2location tutorial. Plots represent estimated abundance for cell types. The size of the Visium spot in the plots was scaled accordingly to enhance visualization.

### CellPhoneDB and CellSign

We updated the CellphoneDB database to include: (1) extra manually curated protein cell–cell interactions (*n* = 1,852 interactions) and (2) cell–cell interactions involving non-protein ligands such as steroid hormones and other small molecules (*n* = 194). For the latter, we used the last bona fide enzyme in the biosynthesis pathway (Supplementary Table 11a,b).

To retrieve interactions between supporting and other cell populations identified in our gonadal samples, we used an updated version of our CellPhoneDB^34,71^ (https://github.com/ventolab/CellphoneDB) approach described in ref. ^72^. In short, we retrieved the interacting pairs of ligands and receptors meeting the following requirements: (1) all the protein members were expressed in at least 10% of the cell type under consideration; and (2) at least one of the protein members in the ligand or the receptor was a differentially expressed gene, with an adjusted *P* value below 0.01 and a log_2_ fold change above 0.2. To account for the distinct spatial location of cells, we further classified the cells according to their location in the developing ovaries (outer cortex, inner cortex, medulla) as observed by Visium and smFISH. We filtered cell–cell interactions to exclude cell pairs that do not share the same location.

Furthermore, we added a new module to the database called CellSign that links receptors in CellphoneDB to their known downstream TF. To build CellSign, we have manually mined the literature to identify TFs with high specificity for an upstream receptor and recorded the relevant pubmed reference number (Supplementary Table 11c). We used this database to link our CellPhoneDB results to the relevant downstream TFs, which were derived from our TF analysis.

### TF analysis

To prioritize the TF relevant for a cell state in a human lineage, we integrated three measurements: (1) expression levels of the TF and (2) the activity status of the TF measured from (2a) the expression levels of their targets (described below in TF activities derived from scRNA-seq) and/or (2b) the chromatin accessibility of their binding motifs (described below in TF motif activity analysis from scATAC-seq). Plots in main figures include TFs meeting the following criteria: (1) TF was differentially expressed, with log_2_ fold change greater than 0.5 and adjusted *P* < 0.01 and (2) TF was differentially active, with log_2_ fold change greater than 0.75 and adjusted *P* < 0.01 in at least one of the TF activity measurements (2a/2b). For mouse and macaque, we performed differential expression analysis only and compared the results to the orthologous TF in humans.

#### TF differential expression

We computed differential expression using the one-sided Wilcoxon Rank Sum test implemented in the FindAllMarkers function with Seurat v.3.2.2, in a one-versus-all fashion.

#### TF activities derived from scRNA-seq

We estimated protein-level activity for human TFs as a proxy of the combined expression levels of their targets. Target genes were retrieved from Dorothea^73^, an orthogonal collection of TF targets compiled from a range of different sources. Next, we estimated TF activities for each cell using Viper^74^, a GSEA-like approach, as implemented in the Dorothea R package and tutorial^75^. Finally, to identify TF whose activity was upregulated in a specific cell type, we applied the Wilcoxon Rank Sum test from Seurat onto the *z*-transformed ‘cell × TF’ activity matrix in a one-versus-all fashion.

#### TF motif activity analysis from scATAC-seq

TF motif activities were computed using chromVar^76^ v.1.12.2 with positional weight matrices from JASPAR2018 (ref. ^77^), HOCOMOCOv10 (ref. ^78^), SwissRegulon^79^, HOMER^80^. chromVar returns a matrix with binding activity estimates of each TF in each cell, which we used to test for differential TF binding activity between cell types in a one-versus-all fashion with Wilcoxon Rank Sum test (FindAllMarkers function in Seurat).

### Reporting summary

Further information on research design is available in the Nature Research Reporting Summary linked to this paper.

## Online content

Any methods, additional references, Nature Research reporting summaries, source data, extended data, supplementary information, acknowledgements, peer review information; details of author contributions and competing interests; and statements of data and code availability are available at 10.1038/s41586-022-04918-4.

## Supplementary information

Supplementary Information Supplementary Notes 1–7 and reference.

Reporting Summary

Supplementary Tables Supplementary Tables 1–11 and legends to the tables.

## Source data

Source Data Extended Data Fig. 1.

Source Data Extended Data Fig. 8.

## Data Availability

Datasets are available from ArrayExpress (www.ebi.ac.uk/arrayexpress), with accession numbers E-MTAB-10551 (human scRNA-seq), E-MTAB-10570 (human scATAC-seq), E-MTAB-11708 (human snRNA-seq/scATAC-seq multiomics), E-MTAB-10589 (human Visium) and E-MTAB-11480 (Mouse scRNA-seq). Multiplexed smFISH images are available from BioStudies (www.ebi.ac.uk/biostudies), with accession number S-BIAD393. All data are public access. scRNA-seq datasets to reproduce UMAPs and dot plots can be accessed and downloaded through the web portals www.reproductivecellatlas.org. External datasets for macaque (GSE149629), mouse (GSE136220 and GSE136441) and human (GSE86146) gonads are available through their respective accessions from GEO. External raw sequencing data from human developing tissues are available from ArrayExpress (E-MTAB-7407, E-MTAB-8901, E-MTAB-8581, E-MTAB-0701, E-MTAB-9801) or GEO (GSE141862). Source data are provided with this paper.

## Extended data

are available for this paper at 10.1038/s41586-022-04918-4.

## Code availability

All the code used for data analysis is available at https://github.com/Ventolab/HGDA.
