# Supplementary material for: Single-cell roadmap of human gonadal development
Source: Nature. 2022 Jul 6;607(7919):540–7. doi: 10.1038/s41586-022-04918-4 (PMC9300467; doi:10.1038/s41586-022-04918-4)
Supplement: Supplementary file 1 — Supplementary Notes 1–7 and reference. [file 41586_2022_4918_MOESM1_ESM.pdf]

nature portfolio

https://doi.org/10.1038/s41586-022-04918-4

Supplementary information

Single-cell roadmap of human gonadal development

In the format provided by the authors and unedited

Supplementary Information for
Single-cell roadmap of human gonadal development

Luz Garcia-Alonso †, Valentina Lorenzi †, Cecilia Icoresi Mazzeo, João Pedro Alves-Lopes, Kenny Roberts, Carmen Sancho-Serra, Justin Engelbert, Magda Marečková, Wolfram H Gruhn, Rachel A Botting, Tong Li, Berta Crespo, Stijn van Dongen, Vladimir Kiselev, Elena Prigmore, Mary Herbert, Ashley Moffett, Alain Chedotal, Omer Bayraktar, Azim Surani, Muzlifah Haniffa, Roser Vento-Tormo*

Table of Contents

Supplementary Notes **2**

Supplementary Note 1. Annotation of main cell lineages. 2

Supplementary Note 2. Cross-species comparison of cell types. 4

Supplementary Note 3. Characterisation of germ cells. 5

3.1. Annotation of cell types 5

3.2. Identification of key transcription factor (TF) regulatory programs 6

Supplementary Note 4. Annotation of early gonadal somatic cells. 6

Supplementary Note 5. Annotation of supporting PAX8+ cells (sPAX8s). 9

Supplementary Note 6. Characterisation of the two waves of human granulosa cells. 10

6.1. Annotation of sell types 10

6.2. Human-mouse comparison 11

6.3. Identification of key TF regulatory programs 11

6.4. Germ-supporting cellular crosstalk 12

Supplementary Note 7. Characterisation of testis-specific resident macrophages. 13

Supplementary References **15**

Supplementary Notes

Supplementary Note 1. Annotation of main cell lineages.

The sexually undifferentiated gonadal ridge (pregursor of the ovary and testis) emerges from the thickening of the mesothelial layer of the peritoneum (i.e. the coelomic epithelium) of the mesonephros. Around 6 post-conceptional weeks (PCWs), the bipotent gonad adopts either female (default program) or male fate, being the first of the reproductive organs to differentiate. Here, we acquired developing human gonadal (i.e., gonadal ridge, testes and ovaries) and extragonadal tissue (i.e., mesonephros, mesonephric ducts and paramesonephric ducts) in the first and second trimester of gestation (n=55) ranging from 6 post-conceptional weeks (PCWs; Carnegie Stage CS16) to 21 PCWs **(Fig. 1a-b, Supplementary Table 1-3)**. Samples were classified as female (n=33) or male (n=22) based on karyotype and expression of male-specific marker genes (i.e., *SRY, RPS4Y1* and *DDX3Y*).

We analysed these samples using multiple genomics methods: i) single-cell RNA sequencing (scRNA-seq), ii) single-cell accessible chromatin sequencing (scATAC-seq), iii) combined single nuclei RNA and ATAC sequencing (snRNA-seq/scATAC-seq) and iv) spatial transcriptomics (10X Genomics Visium, **Extended Data Fig. 1a-d, 2a-c)**. We also profiled single-cell transcriptomes from murine gonadal and extragonadal tissue around the time of sex determination: embryonic days (E) 10.5, 11.5 and 12.5 **(Extended Data Fig. 1a-d, 2a-c)**. Human gonadal and extragonadal tissues were loaded in the same channel of the 10x Genomics chip. For selected human and mouse samples, we separated gonadal from extragonadal tissue and loaded them in different 10x Genomics channels **(Extended Data Fig. 3a)**.

Human and mouse scRNAseq data from male and female samples were analysed separately using a state-of-the-art pipeline (see method *Downstream scRNA-seq analysis*). Mouse scRNA-seq was combined with a publicly available dataset including later gestational stages and postnatal samples (E11.5 to postnatal day (P) 5) to cover equivalent ovarian developmental landmarks such as folliculogenesis. Altogether, after quality control, we recovered 347,709 (human) and 63,929 (mouse) single-cell gene expression profiles that we integrated using scVI to correct for batch effects (**Fig. 1c, Extended Data Fig. 1a-d, 2a-c**). We identify sixteen main populations based on their expression of known markers (**Extended Data Fig. 1d, Supplementary Table 3**). Germ (*DAZL*+), early supporting (*GATA4*+, *WNT6*+), pre-granulosa (female-specific supporting cells) (*WNT6*+, *FOXL2*+) and Sertoli (males-specific supporting cells) (*WNT6+, SOX9*+) are exclusive to the gonads **(Extended Data Fig. 3a)**. Epithelial cells (*EPCAM*+) are only observed in extragonadal tissue, likely from the mesonephric tubules or the mesonephric (i.e., Wolffian) and paramesonephric (i.e., Müllerian) ducts **(Extended Data Fig. 3a)**. Nervous system cells are mostly in samples at CS16-18 (=<7 PCWs). Mesenchymal (*PDGFRA*+), mesothelial (*UPK3B+,* including the coelomic epithelium), endothelial (*PECAM*+), perivascular (PV; *ACTA2*+), erythroid (*HBA1*+) and immune (*CD45*+) subsets are common to both gonads and extragonadal tissue **(Extended Data Fig. 3a)**. The major cell types identified in our analysis have been described in another single-cell transcriptomic study of human gonads[1]**,** however, the highly granular annotation of somatic cell lineages is only resolved in our dataset

(332,862 versus 677 somatic cells analysed in our and Li *et at.* datasets[1], respectively) **(Extended Data Fig. 1e)**.

The gonadal ridge emerges from the coelomic epithelium on the ventral surface of the mesonephros, forming a continuous with the mesonephros. As the gonad develops, it gradually separates from the mesonephros. To define the full map of somatic cell populations specific to the human gonad, we further characterised and located mesenchymal and mesothelial (i.e., coelomic epithelial) subsets by looking at their markers and mapping the cells in the tissue **(Extended Data Fig. 3a,d)**. Mesenchymal and mesothelial subsets display mutually exclusive expression of the transcription factors *GATA4/LHX9/ARX/NR5A1* (i.e., gonadal markers) or *GATA2* **(Extended Data Fig. 3d)**. Using multiplexed smFISH, we find that the expression of *GATA4* is exclusive to the gonads, including the gonadal coelomic epithelium (*UPK3B+*), while expression of *GATA2* is restricted to extragonadal tissue at CS17 (~6 PCWs) in humans **(Extended Data Fig. 3c)**. Visium analysis shows that *GATA4/LHX9/ARX* expression is gonad-specific in later stages (8-12 PCWs), while *GATA2* expression is restricted to extragonadal tissue **(Extended Data Fig. 3e)**. Accordingly, deconvolution of Visium spots with cell2location locates mesenchymal *GATA4+* subsets (i.e., gonadal interstitial (Gi), ovarian interstitial (Oi), testis interstitial (Ti) and male Fetal Leydig cells) inside the gonad, while mesenchymal *GATA2+* cells are outside the gonad **(Extended Data Fig. 3b)**. Separation by microdissection of the gonad from the extragonadal tissue followed by single cell transcriptomics, further confirmed that *Gata4+/Lhx9+/Arx+* cells are exclusive to the gonadal tissue from E10.5 in mice, while *Gata2+* is restricted to extragonadal tissue **(Extended Data Fig. 3a)**. This is in line with *GATA4* expression being required for differentiation of the genital ridge and the initial thickening of the coelomic epithelium of the gonadal primordium, the suggested precursor of the supporting and interstitial/steroidogenic lineages of the gonad[2]. The transcription factor *WT1,* a classical gonadal marker required in the mouse for the formation of the gonadal ridge, is broadly expressed in extragonadal subsets (e.g., mesenchymal *GATA2+* and coelomic epithelium *GATA2+*, **Extended Data Fig. 3d**) in both human and mouse. *NR5A1*, another transcription factor key for the establishment of the genital ridge[3], is downregulated by differentiated ovarian and testicular interstitial cells. Hereafter, we use *GATA4+* to label mesenchymal and coelomic epithelial cells residing in the gonad.

To gain insights into the chromatin landscape that shapes developing gonadal cells in humans, we profiled 96,174 cells with single-cell open chromatin sequencing (10x scATAC-seq) and 40,742 cells with combined single-nucleus RNA and ATAC sequencing (snRNA-seq/scATAC-seq) (**Fig. 1c, Extended Data Fig. 2, Supplementary Table 1-2**). We integrated the ATAC modalities from both technologies in the same manifold for each biological sex, females and males, separately (**Fig. 1c, Extended Data Fig. 2b,** see Methods *Alignment, quantification, and quality control of ATAC data,* **Extended Data Fig. 2a**). Briefly, we first aggregated unimodal and multimodal scATAC-seq reads from all the samples of each sex and called the peaks using our in-house *cellatac* tool. The resulting male or female matrices were corrected for sample effect using Harmony. Finally, we transferred cell annotation labels from scRNA-seq to scATAC-seq[4] from matched individuals (**Extended Data Fig. 2c**). To validate the transferred cell identity, we manually annotated snRNA-seq cell barcodes from the multimodal snRNA-seq/snATAC-seq using bona fide markers and visualised the annotation on the ATAC manifold (**Extended Data Fig. 2d**). This

approach enabled us to align the two omics modalities and identify the same cell states defined by the scRNAseq analysis (**Fig. 1c)**.

Supplementary Note 2. Cross-species comparison of cell types.

We compared the transcriptional signatures of the main cell types identified in humans to those of their murine counterparts considering all developmental stages combined (**Extended Data Fig. 1f**). We trained a Support Vector Machine (SVM) classifier on human scRNA-seq dataset and projected the cell type annotations onto the mouse scRNA-seq datasets. Predicted annotations based on the classifier were compared to "ground truth" annotations assigned to cells in the mouse datasets manually using marker genes. The details of our workflow are specified below:

- (i) Gene ID harmonisation and filtering: Mouse gene identifiers were converted to human genes using ENSEMBL Biomart multi-species comparison filter. Genes with multiple mappings were discarded. Additionally, genes associated with the cell cycle were removed to avoid biases.
- (ii) Human training data: Human cell types were downsampled to the cell type with the lowest number of cells to obtain a balanced dataset. 75% of the data were used for training the model and 25% of the data were used to test the model. Raw counts were normalised and log-transformed. The 300 most highly variable genes were selected as features for model training.
- (iii) Model training with the human dataset: We then trained an SVM classifier (sklearn.svm.SVC) on the human training data with 5-fold cross validation. We evaluated the performance of the model on the human test data by reporting the precision, recall and F1-score per cell type label.
- (iv) Model predictions on the mouse dataset: The trained model was then used to project the human cell type labels onto the mouse datasets, after gene ID harmonisation. We obtained a predicted probability value that each cell in the mouse datasets corresponds to every given human cell type annotation. The estimated probabilities between human-mouse matching cell types were calculated and used as a similarity score. The probabilities were visualised with boxplots, where the y-axis represents the predicted probabilities of the SVM model, and the x-axis the cell types. As an example, in the human-mouse boxplot for endothelial, for each cell that was annotated as endothelial in mouse we show the probability of that cell being called endothelial by the SVM model trained on human data.

When comparing the main cell lineages (**Extended Data Fig. 1f**) between human and mouse, our results show great transcriptional similarity for the majority of cell lineages. Notable exceptions include the early supporting and gonadal mesenchymal lineages in both sexes, as well as pre-granulosa cells in females (median prediction probability < 0.4). To verify the robustness of our results, we repeated the analyses by restricting the time windows to: (i) time around sex determination: 6-7 PCWs in humans, E11.5 in mice; and (ii) following sex differentiation in testis and gonads: 8-14 PCWs in humans, E12.5 in mice. For the sex-determination time window, we considered males and females together, while for the later time window we performed the label transfer separately for ovaries and testes. Somatic gonadal cells consistently display the lowest predicted probability, regardless of the

considered developmental time window, indicating that the divergence in the transcriptomic profile between human and mouse is maintained at all stages.

Supplementary Note 3. Characterisation of germ cells.

3.1. Annotation of cell types

During fetal development, primordial germ cells (PGCs) differentiate into pre-spermatogonia in testis or oogonia and oocytes in ovaries. Pre-spermatogonia are arrested in mitosis while oogonia enter meiosis asynchronously. After synapsis and recombination of homologous chromosomes, oogonia are arrested at the diplotene stage of the first meiotic prophase. At the oogonia-oocyte transition, these upregulate the TF *FIGLA* to induce granulosa cells to surround the oocyte and form primordial follicles, which will remain mostly quiescent until reproductive age.

To comprehensively characterise the process of germ cell differentiation in humans, we re-analysed the transcriptomic profile of the germ cells (**Extended Data Fig. 4a-b**). To annotate the resulting germ cell clusters, we combined: (i) the expression of known markers (**Extended Data Fig. 4c**) (ii) the real developmental time at which these cells appear (**Extended Data Fig. 4a,d**) (iii) pseudotime trajectory (**Extended Data Fig. 4e**), and (iv) co-expression from imaging RNAscope (**Extended Data Fig. 5f-g**). Our analysis resolved five major germ cell states:

1. <u>PGCs</u>: PGCs express *NANOS3* and pluripotency markers (*POU5F1+, NANOG+*). They are found at all stages of development, but their proportion decreases with fetal age, representing 100% of total germ cells at 6-7 PCWs and dropping to 12% and 40% at 18-21 PCWs in human ovaries and testis, respectively. In human fetal ovaries, PGCs are enriched in the outer cortex at least until 19 PCWs (the latest stage analysed by Visium).

2. <u>Fetal Germ Cells (FGCs)</u>: FGCs downregulate pluripotency genes (*NANOG-)* and upregulate canonical germ cell markers (*DDX4+, DAZL+*). Trajectory analysis places this population between PGCs and oogonia (females) or pre-spermatogonia (males).

3. <u>Oogonia</u>, including pre-meiotic (*DAZL+/STRA8+*; 3a) and those initiating meiosis (*DAZL+/SYCP1+*; 3b). Oogonia are ovary-specific, emerge around 11 PCWs and account for 50% of the germ cells until 21 PCWs in humans. Trajectory and spatial analyses place this population between FGC and oocytes.

4. <u>Pre-oocytes</u> (*FIGLA+*; 4a) <u>and primary oocytes</u> (*FIGLA+/GDF9+*; 4b). Pre-oocytes are apparent by 14 PCWs, while primary oocytes emerge around 16 PCWs. These subsets increase in frequency as the ovary develops, accounting for 30% of germ cells in 18-21 PCWs in humans. Trajectory analysis places this population as the terminal state of the female germ cell differentiation in the studied developmental window. Spatial analysis locates oocytes in the inner cortex of developing human ovaries.

5. <u>Pre-spermatogonia</u> (*PIWIL4+*): Pre-spermatogonia appear in the human developing testis around 11 PCWs and increase to nearly 60% by 18-21 PCWs. Trajectory

analysis places this population as the terminal state of the male germ cell differentiation in the studied developmental window.

3.2. Identification of key transcription factor (TF) regulatory programs

Next, we set to identify the TFs regulating germ cell development in humans. For this purpose, we reanalysed the scATAC-seq data of the human germ cells (**Extended Data Fig. 5a**) and transferred cell annotation labels from scRNA-seq to scATAC-seq[4] in matching individuals (**Extended Data Fig. 5b**). To validate the transferred cell identities, we annotated snRNA-seq cell barcodes from the multimodal snRNA-seq/snATAC-seq data using the markers described before, and visualised the annotations on the ATAC manifold (**Extended Data Fig. 5c**). This approach enabled us to align the two omic modalities and resolve the same states identified in our transcriptomics analysis (**Extended Data Fig. 4a**).

The aligned multimodal scRNA-seq/scATAC-seq germ cell atlas offers a unique opportunity to investigate the TFs mediating the differentiation from PGCs into either pre-spermatogonia in males or oogonia in females. Here, we combined TF expression and activity measurements, the latter estimated from (i) the expression of consensus targets; and/or (ii) the chromatin accessibility of their binding sites (see Methods *Transcription Factor analysis*). We prioritised those TF that were differentially expressed and activated in each human germ cell state (**Extended Data Fig. 5e**).

To study the expression pattern of these TF during PGC differentiation in other mammals, we integrated our human and mouse transcriptomic atlas with scRNA-seq data from mouse testicular and ovarian[5,6] and macaque ovarian[7] germ cells covering analogous developmental stages (**Extended Data Fig. 4a**). We resolved the same cell states in all species using canonical markers (**Extended Data Fig. 4c**). To compare the expression dynamics of the prioritised TFs among humans, macaques and mice we used differential expression analysis (i.e., Wilcoxon Rank Sum test) and retrieved the significantly overexpressed TFs in each germ cell state (log2-fold change > 0 and FDR < 0.05) across species. TFs with conserved expression dynamics (i.e. upregulated by the same germ cell state in all species) are highlighted with bold-coloured labels in **Extended Data Fig. 5e**.

Overall, our multiomics analysis of germ cell differentiation builds on previous work profiling the transcriptome of human germ cells[8–10], and reveals novel TFs involved in germ cell differentiation (**Extended Data Fig. 5e**). Our cross-species comparison highlights a conserved TF network in primates with some differences from their murine counterparts, in agreement with previous murine-human comparisons of primordial germ cells[8] and spermatogonia[11]. We define novel TFs in humans at the meiotic stage, including *ZIC1* (involved in retinoic acid production), *DMRTB1* and HOX factors (*HOXA1*, *HOXD8*). A role for HOX factors in oocyte maturation has been postulated previously[12,13], but their potential activity in oogenesis is unexplored. Our work also reinforces the relevance of development in postnatal diseases. For example, our analysis highlights the conserved activation of *TP63* in oocytes, a gene associated with ovarian insufficiency[14] and, more recently, with ovarian ageing[15].

Supplementary Note 4. Annotation of early gonadal somatic cells.

Sex determination starts with the expression of *SRY*, the Y-linked testis sex-determining TF[16], in the somatic cells arising from the coelomic epithelium of the bipotential gonadal ridge. Expression of *SRY* in the somatic lineage leads to the specification of the supporting Sertoli cells around 6 PCWs in humans[17]. In the absence of *SRY* expression, somatic cells differentiate into supporting pre-granulosa (preGC) cells *via* RSPO1/WNT4-β-catenin signalling[18,19]. In humans, the bipotent somatic cells giving rise to the first wave of male and female supporting cells have not yet been defined.

To resolve the cellular heterogeneity of the gonadal somatic compartment, we reanalysed the transcriptomic profile of the mesothelial (i.e. coelomic epithelium and OSE), supporting (including early supporting, Sertoli and pre-granulosa), and *GATA4*+ mesenchymal cells along all stages in humans and mice (**Fig. 2a-b, Extended Data Fig. 6a**). Additionally, to further resolve the annotation at the time of sex differentiation, we reanalysed these three somatic populations from samples between 6-8 PCWs. We inferred the ontogeny of gonadal somatic cells by combining multiple layers of information: (i) expression of known marker genes and distinctly expressed genes (**Fig. 2b**) (ii) developmental time at which these cells appear (**Fig. 2d**), (iii) pseudotime trajectory inferred by scRNA-seq data (**Fig. 2c, Extended Data Fig. 6b-c**), and (iv) co-expression of markers using multiplexed smFISH (**Extended Data Fig. 6i**). Altogether, we resolved the following cell states at the time of sex determination.

- "<u>Early somatic</u>" cells appear in both XX and XY gonads and are detected in our scRNA-seq dataset as early as 6 PCWs (**Fig. 2d**). Early somatic cells downregulate general mesothelial markers (*UPK3B-, LRRN4-*) but maintain the expression of the TFs characteristic of all the gonadal populations, sharing TFs with the gonadal coelomic epithelium (*GATA4+, LHX9+, NR5A1+*) and the gonadal interstitial lineage (*ARX+, TCF21+*), and upregulate markers characteristic of the supporting lineage (*WNT6+*, **Fig. 2b**). In human samples at 7 PCWs, trajectory analysis places this population as an intermediate state emerging from the coelomic epithelium *GATA4+*, and connecting more differentiated supporting and interstitial cells (**Fig. 2c, Extended Data Fig. 6a**). We do not observe this population in mice gonads at the stages profiled.
- <u>Gonadal interstitial (Gi)</u> cells appear in both XX and XY gonads and are present in our scRNA-seq dataset as early as 6 PCWs. Gi are the first mesenchymal *GATA4+* cells to emerge, and express *COL1A1, DCN and PDGFRA,* as well as TFs characteristic of the gonadal coelomic epithelium (*GATA4+, LHX9+, NR5A1+, ARX+, TCF21+*). Gi are distinct from the coelomic epithelium in that they do not express mesothelial markers (*UPK3B-, LRRN4-*, **Fig. 2d**). Trajectory analysis connects Gi with the "early somatic" population in humans and the coelomic epithelium in mice (**Fig. 2c, Extended Data Fig. 6b-c**). Gi proportions decrease around 9 PCWs, when interstitial cells that are ovarian-specific (i.e., ovarian interstitial (Oi)) or testis-specific (i.e., testicular interstitial (Ti) and Fetal Leydig) emerge (**Fig. 2d; Extended Data Fig. 3b,d**), suggesting that sexually undifferentiated Gi acquire different states in XX and XY gonads after sex determination.
- <u>Early Supporting Gonadal Cells (ESGCs)</u> appear in both XX and XY gonads. ESGCs are detected from 6 PCWs in humans and E11.5 in mice, and upregulate the supporting marker *WNT6* as well as *DMRT1, CPA2* and *GPR37* (**Fig. 2b**). Human ESGCs upregulate the known stem-cell markers *LGR5*, *TSPAN8 and CXCR4* (**Fig. 2e**). Although mouse ESGCs display basal *Lgr5* expression (**Extended Data Fig.**

6h), *Lgr5* and *Tspan8* are instead upregulated at later stages by the murine ovarian surface epithelium (**Extended Data Fig. 8f**), which is a trend we do not observe in humans (**Fig. 4a**). Thus, LGR5/TSPAN8 mark different populations in humans and mice.

Trajectory inference (human and mice; **Fig. 2c, Extended Data Fig. 6b-c**) and smFISH imaging of key makers (human; **Extended Data Fig. 6i**) are consistent with ESGCs being the progenitors of Sertoli and the first wave of pre-granulosa cells.

  - In the testis, ESGCs are detected around 6-7 PCWs and E11.5 in humans and mice, respectively. XY ESGCs are the first population expressing *SRY* in both species, in agreement with being the progenitors of Sertoli cells. We validated the presence of human ESGCs at 6-7 PCWs in an independent XY gonadal dataset[2021] (**Extended Data Fig. 6d-f**). The fact that ESGCs are present in males for a shorter time than in females agrees with previous experiments demonstrating a finite time window for *SRY* activation[22] and switching from ovarian (default) to testis program.
  - In the ovaries, ESGCs are detected up to 8 PCWs and E12.5 in humans and mice, respectively. At those stages, female ESGCs upregulate *FOXL2*, a canonical marker of pre-granulosa cells, and genes of the RSPO1/WNT4-β-catenin pathway (*WNT4+, RSPO1+, AXIN2+,* **Extended Data Fig. 6g**), characteristic of the first wave of pre-granulosa cells in mice. This is in agreement with ESGCs maturing into pre-granulosa cells. In humans, ovarian ESGCs also upregulate the transcription factor *OSR1+*, characteristic of the first wave of pre-granulosa cells (preGC-I, **Fig. 2e, Extended Data Fig. 6h**).

- <u>Supporting-like PAX8+ cells (sPAX8s)</u> are *PAX8+/EPCAM*^{low} cells that express TFs characteristic of the gonadal coelomic epithelium (*GATA4+, LHX9+, NR5A1+*) and the supporting lineage (*WNT6+*), but downregulate interstitial lineage markers (*ARX+, TCF21+*, **Fig. 2b, Supplementary Note 5**). sPAX8 are present in our scRNA-seq dataset as early as 6 PCWs (**Fig. 2d**). Spatial transcriptomics with Visium and smFISH imaging locate this population in the gonads close to the mesonephric interface (**Fig. 2c, Fig. 3a-c, Extended Data Fig. 7a,f-g**).

Taken together, our analyses align with the hypothesis that *GATA4+* coelomic epithelium gives rise to three main somatic cell lineages: (i) supporting (ESGC, differentiating into Sertoli or preGC-I), (ii) interstitial (Gi, differentiating into Oi or Ti) and, (iii) the previously uncharacterised sPAX8s . Sex determination occurs in ESGCs, which gives rise to the first wave of sex-specific supporting cells in both human and mouse gonads. The table below summarises the distinctive features used to annotate the somatic cells at the time of sex determination, with changing genes highlighted in bold.

**Gonadal Somatic Population**	**Markers and dynamics of gene expression**	**Developmental stages***	**Location** (human)	**Trajectory pseudotime analysis** at 7 PCWs (human) / E11.5 (mouse)
Coelomic epithelium GATA4+	*GATA4+, LHX9+, NR5A1+,* ***ARX+, TCF21+, UPK3B+, LRRN4+***	HUMAN: 6-9 PCWs<br><br>MOUSE: E10.5-14.5	Gonadal surface (Figure 1d)	Initial state

Early somatic	*GATA4+, LHX9+, NR5A1+,* ***ARX+, TCF21+, WNT6+***	HUMAN: 6-7 PCWs<br><br>MOUSE: not found in the analysed stages		Intermediate state.<br><br>Differentiates from the *GATA4+* Coelomic Epithelium
Gonadal interstitial (Gi)	*GATA4+, LHX9+, NR5A1+,* ***ARX+, TCF21+ COLA1A+***	HUMAN: 6-8 PCWs<br><br>MOUSE: E10.5-11.5		Intermediate state.<br><br>Differentiates from Coelomic Epithelium GATA4+ or early somatic (human).
Early sPAX8s	*GATA4+, LHX9+, NR5A1+,* ***WNT6+, PAX8+***	HUMAN: 6-8 PCWs<br><br>MOUSE: E11.5-	Gonads, at the interface with the mesonephros (Figure 4a)	Intermediate state.<br><br>Differentiates from Coelomic Epithelium GATA4+ or ESGCs.
Early Supporting Gonadal Cells (ESGCs)	*GATA4+, LHX9+, NR5A1+,* ***WNT6+, DMRT1+, CPA2+, GPR37+***<br><br>- XY gonads*:* ***SRY+***<br>- XX gonads*:* ***FOXL2+***<br><br>*- Human gonads:* ***LGR5+, TSPAN8+***	HUMAN:<br>XY gonads: 6-7 PCWs<br>XX gonads: 6-8 PCWs<br><br>MOUSE:<br>XY gonads: E11.5<br>XX gonads: E11.5-12.5	Gonadal medulla (Figure 3g-h)	Intermediate state.<br><br>Differentiates from early somatic (human) or coelomic epithelium (mouse).<br><br>Differentiates into Sertoli (XY) or preGC-I (XX).

(*) Note 6 PCWs is the earliest time point included in the human study; while E10.5 is the earliest in the mouse study.

Supplementary Note 5. Annotation of supporting PAX8+ cells (sPAX8s).

We define novel gonadal sPAX8s cells clustering with the supporting lineage (**Supplementary Note 4**). sPAX8s distinctively express *PAX8,* which is a characteristic marker of the epithelial cells from the developing reproductive ducts (i.e., Wolffian and Müllerian ducts; **Fig. 2a-b**). Joint analyses of gonadal (including sPAX8s) and extragonadal epithelial cells (i.e., reproductive duct precursors and mesonephric kidney) reveal independent clustering (**Extended Data Fig. 7b-c**), indicating that they are two independent cellular identities. Accordingly, sPAX8s express low levels of epithelial markers (*EPCAM*^{low}, *KRT19*^{low}) and do not express mesonephric duct markers (*PAX2*-, **Extended Data Fig. 7c**). Instead, sPAX8s share multiple markers with the gonadal supporting population (*GATA4+, LHX9+, NR5A1+, WNT6+*), which are not expressed by the epithelial cells from the reproductive ducts or the mesonephric kidney (**Extended Data Fig. 7c**) Thus, sPAX8s is a population distinct from the epithelial cells of the reproductive ducts and mesonephros, and it is found in both humans and mice.

We used spatial transcriptomics (Visium) and multiplexed smFISH to trace sPAX8s population in different developmental stages. Early sPAX8s (*PAX8+/EPCAM*^{low}) around ~ 7 PCWs (CS19-CS20) form a gradient of expression in the gonads, with higher concentration at the gonadal-mesonephros interface and lower concentration in the cortex (**Fig. 3a; Extended Data Fig. 7d**). Late sPAX8s (*PAX8+/EPCAM*^{low}) appear at ~8 PCWs in males (**Fig. 2d)**, and, in the testis, are in contact with the Sertoli cells at the poles of the developing

testis cords (**Fig. 3b** zoomed-in gonadal section**, Fig. 3c, Extended Data Fig. 7f**). This is different from the location of the epithelial cells (PAX8+/EPCAM^{high}) in the developing Wolffian ducts outside the gonad (**Fig. 3b**; whole gonadal section, **Fig. 3c**). Epithelial cells of the Wolffian ducts are outside the gonad and do not establish contact with the developing testis cords. After 8 PCWs in humans, this population is residual in females (**Extended Data Fig. 7g**). This result is in keeping with the presence of a rudimentary *rete ovarii* at this location early in development; this degenerates at later stages [23].

Supplementary Note 6. Characterisation of the two waves of human granulosa cells.

6.1. Annotation of sell types

To annotate the supporting pre-granulosa cell states in humans we considered: (i) the expression of *bona fide* marker genes (**Fig. 4a**), (ii) the developmental time at which these cells appear (**Fig. 2d**), and (iii) their spatial coordinates, as inferred by integrating single-cell transcriptomics with Spatial Transcriptomics (Visium) (**Fig. 4b**).

- Pre-granulosa cells of the 1st wave (preGC-I) are the first ovarian-specific supporting cells that emerge around early 8 PCWs (**Fig. 2d**). They are transcriptionally similar to the bipotent ESGCs but upregulate the canonical marker of pre-granulosa cells (*FOXL2*+). preGC-I also expresses *WNT4* and *RSPO1*, both genes upstream regulators of the RSPO1/WNT4-β-catenin pathway, and the downstream canonical WNT target *AXIN2* (**Fig. 4a**). Human preGC-I are steroidogenic, as shown by the upregulation of enzymes involved in estrogen biosynthesis, (*HSD17B6+*, *CYP19A1+)*, and the TF *OSR1+* (**Fig. 4a**). Spatial transcriptomics analysis in human ovaries between 11 and 19 PCWs shows that preGC-I become restricted to the ovarian medulla as the ovary differentiates (**Fig. 4b**). This is a likely consequence of the novel 2nd wave of pre-granulosa cells proliferating in the cortex, spatially constraining preGC-I to the medulla.

- Ovarian Surface Epithelium (OSE), is a mesothelial population (*UPK3B*+) specific to the developing ovaries that expresses *IRX3*. In humans, OSE is present from late 8 PCWs, when the number of the gonadal coelomic epithelium *GATA4*+ cells decline (**Fig. 2d**). Spatial transcriptomics analysis maps OSE cells to the outer cortex of the ovaries. OSE displays different markers between human and mouse (**Fig. 4b**). Human OSE distinctively upregulates, among others, the transcription factor *LHX2*, proposed as a marker of OSE in humans[24] (**Fig. 4a**). In contrast, mouse OSE lacks *Lhx2* expression but upregulates the stem cell markers *Lgr5* and *Tspan8* (**Extended Data Fig. 8f**), both characteristic of human ESGCs instead (**Fig. 4a**).

- Pre-granulosa cells of the 2nd wave (preGC-II). Emerging with the establishment of the OSE, we define two subsets of preGC-IIa/b that share expression of *LHX2* with OSE, but downregulate *RSPO1/WNT4* genes.
  - PreGC-IIa co-appear with OSE at mid 8 PCWs (**Fig. 2d**). They express the retinoic acid inhibitor *CYP26B1* (meiosis inhibitor) and low levels of the

granulosa marker *FOXL2* (**Fig. 4a, Extended Data Fig. 8f**). Spatial transcriptomics analysis co-localises preGC-IIa with OSE cells in the outer cortex of the ovaries (**Fig. 4b**).

- PreGC-IIb appear around 11 PCWs (**Fig. 2d**). This subset is transcriptomically similar to preGC-IIa, but expresses the oogenesis activation marker *BMP2* and higher levels of *FOXL2* (**Fig. 4a, Extended Data Fig. 8f**). Spatial transcriptomics analysis localises preGC-IIb in the inner cortex, between cortical preGC-IIa and medullary preGC-I (**Fig. 4b**).

- Developing or follicular granulosa cells upregulate retinol dehydrogenase (RDH10), the NOTCH receptors (*NOTCH2*+ and *NOTCH3*+) and their downstream folliculogenesis effectors (*HES1+, HEY1+, HEY2+, HEYL+ and HES4+*), and additional receptors whose cognate ligands are specifically expressed by primary oocytes (**Fig. 4a,d**). Developing granulosa cells co-appear in the inner cortex with primary oocytes (**Fig. 4b, Extended Data Fig. 5f-g**) around 17 PCWs (**Fig. 2d, Extended Data Fig. 4d**).

Transcriptional similarity, pseudotime analysis and the spatio-temporal co-appearance (i.e., at 8 PCWs in the ovarian medulla) strongly support that the first wave of granulosa cells (preGC-I) in humans originates from ESGCs upregulating RSPO1/WNT4-β-catenin pathway. After sex determination, in developing ovaries, the coelomic epithelium *GATA4*+ activates a set of TFs (including *LHX2*) and differentiates into OSE. OSE initiates a 2nd wave of *LHX2*+ cortical granulosa cells (preGC-II) independent of RSPO1/WNT4-β-catenin, likely restricting preGC-I to the ovarian medulla. In keeping with OSE as the progenitor of 2nd wave of pre-granulosa cells, OSE has a higher proportion of cycling cells (i.e., at S or G2/M stages) than preGC-II (27% versus 3%).

6.2. Human-mouse comparison

The spatiotemporal dynamics of human preGC agrees with the two waves of pre-granulosa cells described in mice[6,25]. Yet the markers defining these populations are distinct between species (**Fig. 4a, Extended Data Fig. 8f**). Using the cross-species comparison workflow described in **Supplementary Note 2**, we compared the transcriptomic profiles of human female supporting cells to their murine counterparts (**Extended Data Fig. 8a**). We computed the predicted probabilities of the label transfer from human to mouse ovaries around the time of the second wave of preGC cells (8-16 PCWs human, E12.5-E16.5 mouse) and around the time of folliculogenesis (17-21 PCWs human, E18.5-P5 mouse). Coelomic epithelium *GATA4+* is consistently predicted with high probability across species. On the other hand, ESGCs, preGC-I, OSE and granulosa are consistently poorly predicted across species (median predicted probability < 0.4), suggesting a divergent transcriptomic profile at all developmental stages.

6.3. Identification of key TF regulatory programs

We used the same approach as described in **Supplementary Note 3.2** to prioritise the TFs relevant for granulosa-cell differentiation. First, we reanalysed the human scATAC-seq data of the supporting lineage and resolved the same cell states identified in our human scRNA-seq analysis (**Fig. 2a, Extended Data Fig. 8b-c**). Second, we used the aligned

multimodal scRNA-seq/scATAC-seq human data to estimate expression and activity of the TFs on each supporting cell state (**Extended Data Fig. 8d**). Third, we combined the expression and activity measurements to prioritise those TF that were differentially expressed and active along the different granulosa states (**Fig. 4c**).

To investigate the conservation in the dynamics of these TF during 1st and 2nd wave of granulosa, we integrated our atlas with mouse and macaque ovarian scRNAseq datasets covering analogous developmental stages (**Fig. 2a, Extended Data Fig. 8e-f**). We resolved the same cell states in all species using canonical markers and developmental stage information, and compared the expression dynamics of the prioritised TF between humans, macaques and mice. Significantly overexpressed TFs in each granulosa state (log2-fold change > 0 and FDR < 0.05) were estimated with differential expression analysis (i.e., Wilcoxon Rank Sum test). TFs with conserved expression dynamics are highlighted with bold-coloured labels in **Fig. 4c**.

6.4. Germ-supporting cellular crosstalk

In developing ovaries, there is a spatiotemporal gradient of co-differentiating granulosa (**Fig. 4b**) and germ cells (**Extended Data Fig. 5f-g**). To investigate the communication between granulosa and germ cells in the different human ovarian microenvironments (i.e., outer cortex, inner cortex, medullary region and follicles), we updated CellPhoneDB to v4 (**Extended Data Fig. 8g**). This updated version of CellPhoneDB doubles the number of manually curated interactions, and includes interactions mediated by small molecules, such as steroid hormones. In addition, we developed CellSign, a novel extension of the database that links receptors with their downstream TFs, as assessed by manual curation (see *Methods CellPhoneDB and CellSign*). TFs are used as sensors of ligand-receptor binding, allowing us to prioritise functional cell–cell interactions that affect downstream signalling.

- In the <u>outer cortex</u> microenvironment, PGC co-locate with coelomic epithelium or the OSE and preGC-IIa **(Extended Data Fig. 5f-g, Fig. 4b)**. These cortical supporting cells are a major source of paracrine factors required for the chemotaxis and survival of PGC, including *BMP4* (coelomic epithelium) and *KITLG* (preGC-IIa), with *STAT3* downstream of *KIT* active in PGC (**Fig. 4d, Extended Data Fig. 8h**). There is also a unique composition of extracellular matrix (ECM) proteins that potentially mediates adhesion of PGC to supporting cells in the outer cortex **(Extended Data Fig. 8i)**. PGCs specifically express collagen IV (*COL4A1/COL4A2*), which is replaced by collagens VI when oogonia become responsive to retinoic acid.

- In the <u>inner cortex and medullary</u> microenvironments, preGC-IIb and preGC-I support oogonia differentiation **(Extended Data Fig. 8k-l, Fig. 4b)**. PreGC-I upregulate enzymes involved in estrogen production, *HSD17B6* and *CYP19A1*, suggesting that they may be capable of producing estrogen locally for oogonia *STRA8+*, which overexpress the estrogen receptor *ESR1* **(Fig. 4d)**. preGC-IIb express the enzyme *ALDH1A1* involved in retinoic acid synthesis, whose product may potentially bind to their receptor (*RARA)* and transporter (*CRABP2)*, both of them expressed by oogonia *STRA8+* **(Fig. 4d).** Accordingly, with CellSign we detect upregulation of *RARA* transcriptional activity in oogonia *STRA8+* (**Extended Data Fig. 8h**). PreGC-IIb and developing granulosa cells also overexpress *BMP2*, recently shown in mice to activate the oogenesis factor *ZGLP1* [26], which we predict to be

active in oogonia *STRA8+* **(Fig. 4d, Extended Data Fig. 8h)**. Novel interactions involve the parathyroid hormone ligand (*PTHLH*) and its receptor (*PTH1R)*, overexpressed by preGC-IIb and oogonia, respectively, and associated with BMP-2 signalling [27,28] (**Fig. 4d).**

- After 17 PCWs in the inner cortex, primary oocytes are enclosed by flattened granulosa cells to form primordial follicles (**Fig. 4b)**, following bidirectional crosstalk. Germ cells generate a gradient of folliculogenesis factors: PGCs express the Notch inhibitor *DLK1*, while oocytes express Notch activator *JAG1* as well as *GDF9*, *TGFα* (**Fig. 4d)**. Accordingly, with CellSign we detect upregulation of the Notch signalling targets (*HES1+, HEY1+, HEY2+ and HES4+,* **Fig. 4c**). Additional signals from oocytes to supporting cells in follicles include the histamine-*HRH1* and neuregulin-*ERBB4* receptors (**Fig. 4d)**. We uncover novel granulosa to oocyte interactions candidates to mediate successful follicular assembly, such as the Netrin-1 (*NTN1*) and its receptor *DCC* (**Fig. 4d**), that may regulate axon guidance, cell migration and apoptosis. We validated this interaction in human follicles using multiplexed smFISH (**Extended Data Fig. 8j**). Accordingly, *OTX2,* a TF required for cell migration via NTN1-*DCC,* is active in oocytes (**Extended Data Fig. 8h**). There is also a unique composition of ECM proteins that potentially mediates adhesion of the oocytes to the follicular granulosa cells **(Extended Data Fig. 8i).**

Altogether, we provide the main factors required to support germ cell differentiation and survival, together with the upregulated TFs **(Extended Data Fig. 8k)**.

Supplementary Note 7. Characterisation of testis-specific resident macrophages.

To comprehensively characterise the immune compartment of the fetal gonads, we sorted cells from 11 samples using the pan-leukocyte marker CD45 and integrated them with immune cells from the main analyses (**Extended Data Fig. 9a-c**). Our immune dataset defined 19 clusters, including haematopoietic stem cells/multipotent progenitors (HSC/MPP), megakaryocytes and mast cells, B cells and other innate lymphocyte lineages, and myeloid cells (**Fig. 5a**). Cell type identities of gonadal immune cells were assigned based on the expression of known marker genes as well as by transferring labels from a human fetal liver haematopoiesis dataset using a SVM model (**Extended Data Fig. 9d-e**)[29].

Tissue-resident myeloid cells likely play a role in testicular development and function[30,31]. Our analysis revealed that the majority (43%) of gonadal immune cells are macrophages with a tissue-repair phenotype *(LYVE1, FOLR2)* (**Extended Data Fig. 9f-g**). Specific to the developing testes, we also identify *SIGLEC15*+ and *TREM2*+ fetal testicular macrophages (ftM) with an osteoclast-like and microglia-like profile, respectively (**Fig. 5a-b,d**, **Extended Data Fig. 9g-h, Extended Data Fig. 10a**). A distinctive expression pattern of molecules affecting immune cell function in both populations suggests that they may seed an immunoregulatory environment previously described in prepubertal testes (**Extended Data Fig. 9m**)[32,33].

SIGLEC15+ ftM are found in the peritubular spaces surrounding the testis cords, interacting with endothelial and mesenchymal cells via extracellular matrix proteins (**Fig. 5d**, **Extended**

Data Fig. 9l, **Extended Data Fig. 10b**). Their numbers peak ~8-14 PCWs, coinciding with the period of mesonephric endothelial cell migration[34]. We confirmed the developmental window of *SIGLEC15+* ftM by re-analysing data from an independent fetal testes scRNA-seq study (**Extended Data Fig. 10c**)[21]. Endothelial cells from the mesonephros migrate into the developing testes and assemble in the coelomic artery, a process that is required for testis cord formation[34]. This is redolent of findings that osteoclasts contribute to bone angiogenesis via metalloproteinase-9[35]. With such lines of evidence in mind, it is reasonable to speculate that macrophages with an osteoclast-like phenotype might allow for ECM remodelling that is needed in neovascularization processes, such as the formation of the coelomic artery in the fetal testis. Moreover, testicular tissue-resident macrophages in mice have been shown to play a role in controlling vascular and tissue pruning[36].

TREM2+ ftM are the first immune population found mainly inside the forming testis cords (**Fig. 5d, Extended Data Fig. 10d-e**). Although microglia-like cells have been previously identified in association with peripheral nerves in other tissues (skin, gut, sciatic nerve)[37–39], they lack expression of core microglial genes, including *SALL1*. Testicular *TREM2+* ftM have an identical transcriptomic profile to brain microglia, including *SALL1* [40,41]; this probably reflects a shared origin and function. Our integration analysis of myeloid cells from multiple developing organs revealed that *TREM2+* macrophages with a microglia-like signature are also present in the fetal skin (**Fig. 5c**). To further rule out the hypothesis that *TREM2+* ftM could be nerve-associated macrophages, we used markers specific to fetal macrophages with a microglia-like profile (*CD68, P2RY12*) and co-stained a full transversal embryo section with a marker for neural cells (*ELAVL3*). Microglia in the central nervous system (*CD68, P2RY12*) are located close to the *ELAVL3+* expressing cells, but we could not find this pattern for *TREM2+* ftM and fetal skin macrophages (**Extended Data Fig. 10d**). Additionally, interactions resulting from the association of *TREM2+* ftM with Sertoli cells and spermatogonia within the seminiferous tubules suggest that they could play a role in removing apoptotic germ cells (**Extended Data Fig. 9l**).

Supplementary References

1. Li, L. *et al.* Single-Cell RNA-Seq Analysis Maps Development of Human Germline Cells and Gonadal Niche Interactions. *Cell Stem Cell* **20**, 858–873.e4 (2017).
2. Hu, Y.-C., Okumura, L. M. & Page, D. C. Gata4 is required for formation of the genital ridge in mice. *PLoS Genet.* **9**, e1003629 (2013).
3. Luo, X., Ikeda, Y. & Parker, K. L. A cell-specific nuclear receptor is essential for adrenal and gonadal development and sexual differentiation. *Cell* **77**, 481–490 (1994).
4. Stuart, T. *et al.* Comprehensive Integration of Single-Cell Data. *Cell* **177**, 1888–1902.e21 (2019).
5. Mayère, C. *et al.* Single-cell transcriptomics reveal temporal dynamics of critical regulators of germ cell fate during mouse sex determination. *FASEB J.* **35**, e21452 (2021).
6. Niu, W. & Spradling, A. C. Two distinct pathways of pregranulosa cell differentiation support follicle formation in the mouse ovary. *Proc. Natl. Acad. Sci. U. S. A.* **117**, 20015–20026 (2020).
7. Zhao, Z.-H. *et al.* Single-cell RNA sequencing reveals regulation of fetal ovary development in the monkey (Macaca fascicularis). *Cell Discov* **6**, 97 (2020).
8. Tang, W. W. C. *et al.* A Unique Gene Regulatory Network Resets the Human Germline Epigenome for Development. *Cell* **161**, 1453–1467 (2015).
9. Gkountela, S. *et al.* DNA Demethylation Dynamics in the Human Prenatal Germline. *Cell* **161**, 1425–1436 (2015).
10. Guo, F. *et al.* The Transcriptome and DNA Methylome Landscapes of Human Primordial Germ Cells. *Cell* **161**, 1437–1452 (2015).
11. Shami, A. N. *et al.* Single-Cell RNA Sequencing of Human, Macaque, and Mouse Testes Uncovers Conserved and Divergent Features of Mammalian Spermatogenesis. *Dev. Cell* **54**, 529–547.e12 (2020).
12. Adjaye, J. & Monk, M. Transcription of homeobox-containing genes detected in cDNA libraries derived from human unfertilized oocytes and preimplantation embryos. *Mol. Hum. Reprod.* **6**, 707–711 (2000).
13. Villaescusa, J. C., Verrotti, A. C., Ferretti, E., Farookhi, R. & Blasi, F. Expression of Hox cofactor genes during mouse ovarian follicular development and oocyte maturation. *Gene* **330**, 1–7 (2004).
14. Tucker, E. J. *et al.* TP63-truncating variants cause isolated premature ovarian insufficiency. *Hum. Mutat.* **40**, 886–892 (2019).
15. Ruth, K. S. *et al.* Genetic insights into biological mechanisms governing human ovarian ageing. *Nature* **596**, 393–397 (2021).
16. Albrecht, K. H. & Eicher, E. M. Evidence that Sry is expressed in pre-Sertoli cells and Sertoli and granulosa cells have a common precursor. *Dev. Biol.* **240**, 92–107 (2001).
17. Hanley, N. A. *et al.* SRY, SOX9, and DAX1 expression patterns during human sex determination and gonadal development. *Mech. Dev.* **91**, 403–407 (2000).
18. Chassot, A.-A. *et al.* Activation of -catenin signaling by Rspo1 controls differentiation of the mammalian ovary. *Human Molecular Genetics* vol. 17 1264–1277 (2008).
19. Vainio, S., Heikkilä, M., Kispert, A., Chin, N. & McMahon, A. P. Female development in mammals is regulated by Wnt-4 signalling. *Nature* **397**, 405–409 (1999).
20. Chitiashvili, T. *et al.* Female human primordial germ cells display X-chromosome dosage

compensation despite the absence of X-inactivation. *Nat. Cell Biol.* **22**, 1436–1446 (2020).
21. Guo, J. *et al.* Single-cell analysis of the developing human testis reveals somatic niche cell specification and fetal germline stem cell establishment. *Cell Stem Cell* **28**, 764–778.e4 (2021).
22. Hiramatsu, R. *et al.* A critical time window of Sry action in gonadal sex determination in mice. *Development* **136**, 129–138 (2009).
23. Pansky, B. Review of MEDICAL EMBRYOLOGY. (1982).
24. Bowen, N. J. *et al.* Gene expression profiling supports the hypothesis that human ovarian surface epithelia are multipotent and capable of serving as ovarian cancer initiating cells. *BMC Med. Genomics* **2**, 71 (2009).
25. Mork, L. *et al.* Temporal differences in granulosa cell specification in the ovary reflect distinct follicle fates in mice. *Biol. Reprod.* **86**, 37 (2012).
26. Nagaoka, S. I. *et al.* ZGLP1 is a determinant for the oogenic fate in mice. *Science* **367**, (2020).
27. Liu, F. *et al.* Involvement of parathyroid hormone-related protein in vascular calcification of chronic haemodialysis patients. *Nephrology* **17**, 552–560 (2012).
28. Minina, E. *et al.* BMP and Ihh/PTHrP signaling interact to coordinate chondrocyte proliferation and differentiation. *Development* **128**, 4523–4534 (2001).
29. Popescu, D.-M. *et al.* Decoding human fetal liver haematopoiesis. *Nature* **574**, 365–371 (2019).
30. Shechter, R., London, A. & Schwartz, M. Orchestrated leukocyte recruitment to immune-privileged sites: absolute barriers versus educational gates. *Nat. Rev. Immunol.* **13**, 206–218 (2013).
31. Mossadegh-Keller, N. & Sieweke, M. H. Testicular macrophages: Guardians of fertility. *Cell. Immunol.* **330**, 120–125 (2018).
32. Chen, Q., Deng, T. & Han, D. Testicular immunoregulation and spermatogenesis. *Semin. Cell Dev. Biol.* **59**, 157–165 (2016).
33. Meinhardt, A. & Hedger, M. P. Immunological, paracrine and endocrine aspects of testicular immune privilege. *Mol. Cell. Endocrinol.* **335**, 60–68 (2011).
34. Combes, A. N. *et al.* Endothelial cell migration directs testis cord formation. *Dev. Biol.* **326**, 112–120 (2009).
35. Cackowski, F. C. *et al.* Osteoclasts are important for bone angiogenesis. *Blood* vol. 115 140–149 (2010).
36. DeFalco, T. & Bhattacharya, I. Yolk-sac–derived macrophages regulate fetal testis vascularization and morphogenesis. *Proceedings of the* (2014).
37. Ydens, E., Amann, L., Asselbergh, B. & Scott, C. L. Profiling peripheral nerve macrophages reveals two macrophage subsets with distinct localization, transcriptome and response to injury. *Nature* (2020).
38. Kolter, J. *et al.* A Subset of Skin Macrophages Contributes to the Surveillance and Regeneration of Local Nerves. *Immunity* **50**, 1482–1497.e7 (2019).
39. De Schepper, S. *et al.* Self-Maintaining Gut Macrophages Are Essential for Intestinal Homeostasis. *Cell* **176**, 676 (2019).
40. Buttgereit, A. *et al.* Sall1 is a transcriptional regulator defining microglia identity and function. *Nat. Immunol.* **17**, 1397–1406 (2016).
41. Bennett, F. C. *et al.* A Combination of Ontogeny and CNS Environment Establishes Microglial Identity. *Neuron* **98**, 1170–1183.e8 (2018).
