# Supplementary material for: Single-cell roadmap of human gonadal development
Source: Nature. 2022 Jul 6;607(7919):540–7. doi: 10.1038/s41586-022-04918-4 (PMC9300467; doi:10.1038/s41586-022-04918-4)
Supplement: Supplementary file 2 — Reporting Summary [file 41586_2022_4918_MOESM2_ESM.pdf]

nature portfolio

Corresponding author(s): Roser Vento-Tormo

Last updated by author(s): May 17, 2022

Reporting Summary

Nature Portfolio wishes to improve the reproducibility of the work that we publish. This form provides structure for consistency and transparency in reporting. For further information on Nature Portfolio policies, see our Editorial Policies and the Editorial Policy Checklist.

Statistics

For all statistical analyses, confirm that the following items are present in the figure legend, table legend, main text, or Methods section.

n/a	Confirmed	
☐	☒	The exact sample size (*n*) for each experimental group/condition, given as a discrete number and unit of measurement
☐	☒	A statement on whether measurements were taken from distinct samples or whether the same sample was measured repeatedly
☐	☒	The statistical test(s) used AND whether they are one- or two-sided<br>*Only common tests should be described solely by name; describe more complex techniques in the Methods section.*
☐	☒	A description of all covariates tested
☐	☒	A description of any assumptions or corrections, such as tests of normality and adjustment for multiple comparisons
☐	☒	A full description of the statistical parameters including central tendency (e.g. means) or other basic estimates (e.g. regression coefficient) AND variation (e.g. standard deviation) or associated estimates of uncertainty (e.g. confidence intervals)
☐	☒	For null hypothesis testing, the test statistic (e.g. *F*, *t*, *r*) with confidence intervals, effect sizes, degrees of freedom and *P* value noted<br>*Give P values as exact values whenever suitable.*
☒	☐	For Bayesian analysis, information on the choice of priors and Markov chain Monte Carlo settings
☒	☐	For hierarchical and complex designs, identification of the appropriate level for tests and full reporting of outcomes
☒	☐	Estimates of effect sizes (e.g. Cohen's *d*, Pearson's *r*), indicating how they were calculated

Our web collection on statistics for biologists contains articles on many of the points above.

Software and code

Policy information about availability of computer code

Data collection	No software was used for data collection
Data analysis	Alignement, QC and barcode calling of scRNAseq data with Cell Ranger v.3.1.0 (10x Genomics).<br>Alignement, QC and barcode calling of multiomics GEX and ATACseq data with Cell Ranger ARC v.1.0.0 (10x Genomics).<br>Alignement, QC and barcode calling of scATACseq data with Cell Ranger ATAC v.1.2.0 (10x Genomics).<br>Peak calling of scATAC data with custom cellatac available at https://github.com/cellgeni/cellatac<br>Alignement, QC and image processing Visium spatial transcriptomics data with Space Ranger v.1.2.1 (10x Genomics).<br><br>Downstream Analysis was carried out using Python (version 3) with Scanpy v.1.7.0.<br>Batch correction was carried out with scVI v.0.6.8.<br>Marker genes where identified using R v.4.0.3 with SoupX R package v.1.5.0. and Seurat v.3.2.2.<br>Trajectory analysis was done using CellRank package v.1.5.1.<br>ATAC dimensionality reduction and gene activity scores were calcuated with cisTopic v.0.3.0.<br>Cis-co-accessibility networks (CCANs) were estimated using the R package Cicero 69 v.1.3.4.11.<br>Transcription factor motif activities were computed using chromVar v.1.12.2<br>Custom code available at https://github.com/ventolab/HGDA.<br><br>The FACS sorter was controlled with BD FACS DIVA software v.7, and FlowJo v.10.3 was used for analysis.

For manuscripts utilizing custom algorithms or software that are central to the research but not yet described in published literature, software must be made available to editors and reviewers. We strongly encourage code deposition in a community repository (e.g. GitHub). See the Nature Portfolio guidelines for submitting code & software for further information.

Data

Policy information about availability of data

All manuscripts must include a data availability statement. This statement should provide the following information, where applicable:

- Accession codes, unique identifiers, or web links for publicly available datasets
- A description of any restrictions on data availability
- For clinical datasets or third party data, please ensure that the statement adheres to our policy

Datasets are available from ArrayExpress (www.ebi.ac.uk/arrayexpress), with accession numbers E-MTAB-10551 (human scRNA-seq), E-MTAB-10570 (human scATAC-seq), E-MTAB-11708 (human snRNA-seq/scATAC-seq multiomics), E-MTAB-10589 (human Visium) and E-MTAB-11480 (Mouse scRNA-seq). Multiplexed smFISH images are available from BioStudies (www.ebi.ac.uk/biostudies), with accession number S-BIAD393. All data is public access. scRNAseq datasets to reproduce UMAPs and dotplots can be accessed and downloaded through the web portals www.reproductivecellatlas.org.

External datasets for macaque (GSE149629), mouse (GSE136220 and GSE136441) and human (GSE86146) gonads are available through their respective accessions from GEO. External raw sequencing data from human developing tissues is available from ArrayExpress (E-MTAB-7407, E-MTAB-8901, E-MTAB-8581, E-MTAB-0701, E-MTAB-9801) or GEO (GSE141862).

Field-specific reporting

Please select the one below that is the best fit for your research. If you are not sure, read the appropriate sections before making your selection.

- [x] Life sciences
- [ ] Behavioural & social sciences
- [ ] Ecological, evolutionary & environmental sciences

For a reference copy of the document with all sections, see nature.com/documents/nr-reporting-summary-flat.pdf

Life sciences study design

All studies must disclose on these points even when the disclosure is negative.

Sample size	We collected human fetal gonadal tissue from 55 donors. This cohort is equal or larger than previous single-cell transcriptomic atlases of fetal tissues in humans (PMID:33184181; PMID:31597962; PMID:32079746; PMID:33208946), and should be sufficient to capture the main cell types and states in the tissue.<br><br>We collected developing mouse gonads and mesonephros from 23 embryos. Embryos were genotyped to identify the gender. At least 2 males and 2 females (mouse) were collected on any particular day. Specifically, we included 6 males and 3 females at E10.5, 6 males and 2 females at E11.5, and 3 males and 3 females at E12.5. There were no calculations performed to determine sample size. This cohort is equal or larger than previous reference single-cell transcriptomic atlas of fetal tissues in mouse (PMID:30283141), and should be sufficient to capture the main cell types and states in the tissue.
Data exclusions	Seven scRNAseq libraries (all human tissues) were excluded from the final dataset due to low sequencing quality.
Replication	For single cell transcriptomics atlasing, eight tissues were split and processed in parallel to study technical variability. Analysis of technical replicates revealed the same gonadal populations.<br>For spatial transcriptomics, we included two technical replicates (consecutive tissue slides) for each of the five tissues analysed and confirmed replicability of the cell mappings.<br>For high-resolution imaging using RNAScope probes, we performed the analysis on at least two slides from distinct donors. All attempts were successful.
Randomization	Human samples were randomly allocated to this study through (HDBR; http://www.hdbr.org). Human sample collection was based on availability of fetal donors.<br>Mice were not randomized due to practical constraints.<br>Since we aim to characterise the cellular dynamics of the developing gonad, we allocated both human and donor samples into developmental windows based on age. Randomisation is not relevant for this study as we re not comparing disease groups.
Blinding	This study made no comparison between discreet groups for human and mouse individuals, thus binding of investigators was not necessary.

Reporting for specific materials, systems and methods

We require information from authors about some types of materials, experimental systems and methods used in many studies. Here, indicate whether each material, system or method listed is relevant to your study. If you are not sure if a list item applies to your research, read the appropriate section before selecting a response.

Materials & experimental systems

n/a	Involved in the study
☐	☒ Antibodies
☒	☐ Eukaryotic cell lines
☒	☐ Palaeontology and archaeology
☐	☒ Animals and other organisms
☐	☒ Human research participants
☒	☐ Clinical data
☒	☐ Dual use research of concern

Methods

n/a	Involved in the study
☒	☐ ChIP-seq
☐	☒ Flow cytometry
☒	☐ MRI-based neuroimaging

Antibodies

Antibodies used	CD45-BUV395 BD Bioscience 563791 Clone HI30 (RUO) Flow cytometry - index data; 2.5ul:100ul
Validation	CD45-BUV395 Flow cytometry (Routinely Tested) . Flow cytometric analysis of CD45 expression on human peripheral blood lymphocytes (website).

Animals and other organisms

Policy information about studies involving animals; ARRIVE guidelines recommended for reporting animal research

Laboratory animals	We used E10.5, E11.5, E12.5 and E13.5 mouse embryos carrying the Oct4ΔPE-GFP transgene (GOF strain). Mice were housed in specific pathogen-free conditions at a Home Office-approved facility at the University of Cambridge. Mice were maintained with a 12 hour light/ 12 hour dark cycle, with temperature ranging from 20-24°C and humidity of 45-65%.
Wild animals	The study did not involve wild animals
Field-collected samples	The study did not involve samples collected from the field
Ethics oversight	All experimental procedures were carried out in agreement with the project license PE596D1FE issued by the UK Home Office (Animal Welfare Ethical Review Board (AWERB) committee) and carried out in a Home Office designated facility, in accordance with ethical guidelines with the United Kingdom Animals (Scientific Procedures) Act of 1986.

Note that full information on the approval of the study protocol must also be provided in the manuscript.

Human research participants

Policy information about studies involving human research participants

Population characteristics	Human fetal gonads and mesonephros samples were obtained from both males and females, with age ranging between 6-21 post-conception weeks.
Recruitment	Human embryo and fetal samples were obtained from the MRC and Wellcome-funded Human Developmental Biology Resource (HDBR, http:// www.hdbr.org), with appropriate maternal written consent and approval from the Fulham Research Ethics Committee (REC reference 18/LO/0822) and Newcastle & North Tyneside 1 Research Ethics Committee (REC reference 18/NE/0290). The HDBR is regulated by the UK Human Tissue Authority (HTA; www.hta.gov.uk) and operates in accordance with the relevant HTA Codes of Practice.
Ethics oversight	Human embryo and fetal samples were obtained from the MRC and Wellcome-funded Human Developmental Biology Resource (HDBR43, http:// www.hdbr.org), with appropriate maternal written consent and approval from the Newcastle and North Tyneside NHS Health Authority Joint Ethics Committee (08/H0906/21+5). The HDBR is regulated by the UK Human Tissue Authority (HTA; www.hta.gov.uk) and operates in accordance with the relevant HTA Codes of Practice.

Note that full information on the approval of the study protocol must also be provided in the manuscript.

Flow Cytometry

Plots

Confirm that:

☒ The axis labels state the marker and fluorochrome used (e.g. CD4-FITC).

☒ The axis scales are clearly visible. Include numbers along axes only for bottom left plot of group (a 'group' is an analysis of identical markers).

☒ All plots are contour plots with outliers or pseudocolor plots.

☒ A numerical value for number of cells or percentage (with statistics) is provided.

Methodology

Sample preparation	Cells were incubated at 4°C with 2.5ul of antibodies in 1% FBS in DPBS without Calcium and Magnesium (ThermoFisher Scientific, 14190136). DAPI was used for live/dead discrimination.
Instrument	Becton Dickinson (BD) FACS Aria Fusion. For granule experiment we used LSRFortessa FACS analyser (BD Biosciences)
Software	Becton Dickinson (BD) FACS Aria Fusion was controlled using BD FACS DIVA software (version 7) and FlowJo v10.3 was used for analysis.
Cell population abundance	Abundance of CD45 positive and negative fractions for droplet single sequencing was determined by flow cytometry (~1.5% of CD45+ in live, singlet fraction). The purity of these populations was determined by single-cell RNA sequencing.
Gating strategy	Cells isolated for were gated on: live; singlets, and the following cell types sorted using overlapping gates: i) CD45+ ii) CD45-

☒ Tick this box to confirm that a figure exemplifying the gating strategy is provided in the Supplementary Information.
