# Supplementary material for: Single-cell roadmap of human gonadal development
Source: Nature. 2022 Jul 6;607(7919):540–7. doi: 10.1038/s41586-022-04918-4 (PMC9300467; doi:10.1038/s41586-022-04918-4)
Supplement: Supplementary file 3 — Supplementary Tables 1–11 and legends to the tables. [file 41586_2022_4918_MOESM3_ESM.zip › legends for Supplementary Tables 1-11.docx]

# **Supplementary Tables Legends**

**Supplementary Table 1. Extended_Table1.xlsx**

**Metadata of samples.** **(A)** 10x scRNAseq libraries from human donors. **(B)** 10x scRNAseq libraries CD45+ enriched from human donors. **(C)** 10x scATACseq libraries from human donors. **(D)** 10x cell-coupled snRNA/ATACseq multiomic libraries from human donors. **(E)** 10x Visium transcriptomics libraries from human donors. **(F)** 10x scRNAseq libraries from mouse sample. Columns across tables indicate: Sample id = 10x reaction; Donor = donor ID; Stage_PCW = post-conceptional weeks; Stage_CS = Carnegie Stage, if appropiated; Sex = female or male determined by karyotype and sequencing; Library_enrichment = total (no enrichment); CD45- (non-immune fraction); CD45+ (immune fraction); sample_source = center of procedence (L or N); cryo = yes, if cryopreserved; TP = type of pregnancy termination (Med: medical, or Sur: surgical); 10x scRNAseq kit = 5’ reaction (v1 or v2).

**Supplementary Table 2. Extended_Table2.xlsx**

**Quality control of samples.** **(A)** Table containing summary statistics from 10x Cell Ranger 3.1.0 (scRNAseq) and 10x Cell Ranger ARC 1.0.0 (multiomics) for each 10x RNA library in our fetal gonads atlas. **(B)** Table containing summary statistics from 10x Cell Ranger ATAC 1.2 (scRNAseq) and 10x Cell Ranger ARC 1.0.0 (multiomics) for each 10x ATAC library in our fetal gonads atlas.

**Supplementary Table 3. Extended_Table3.xlsx**

**General annotation summary for each scRNAseq sample.** All tables contain the number of cells per cell type and library for (**A**) human female scRNAseq analysis (**B**) human male scRNAseq analysis (**C**) mouse female scRNAseq analysis (**D**) mouse male scRNAseq analysis.

**Supplementary Table 4. Extended_Table4.xlsx**

**Germ cells summary for each scRNAseq sample.** All tables contain the number of germ cell states from each library for (**A**) human female scRNAseq analysis (**B**) human male scRNAseq analysis (**C**) mouse female scRNAseq analysis (**D**) mouse male scRNAseq analysis.

**Supplementary Table 5. Extended_Table5.xlsx**

**TF cross-species multiomic analysis in germ cells.** Table containing the multiple TF measurements in human, mouse and macaque used to prioritise TF relevant for germ cell differentiation. Statistics estimated with the one-sided Wilcoxon Rank Sum test implemented in the FindAllMarkers function in Seurat. Columns across table indicate:

*TF* = transcription factor; *cluster* = cell type; *N_agreement* = number of evidences where the TF is significant in the cell type; *ATAC_PWM* = PWM used to estimate motif enrichment; *RNA_DEGs.avg_logFC* = average log2-Fold change in expression; *RNA_DEGs.pct.1* = % cells expressing the TF in the cell type; *RNA_DEGs.pct.2* = % cells expressing the TF in the remaining cell types of the lineage; *RNA_DEGs.p_val_adj* = adjusted p-value from the differential expression analysis performed with Wilcoxon test; *RNA_DEGs* = 1 if it is a differentially expressed gene; *RNA_TFact.avg_logFC* = average log2-Fold change in activity estimated from Dorothea regulons; *RNA_TFact.p_val_adj* = adjusted p-value from the differential activity analysis performed with Wilcoxon test; *RNA_TFact.Dorothea_score* = Dorothe score for the TF; RNA_TFact = 1 if it is a differentially activated TF; ATAC_TFact.avg_logFC = average log2-Fold change in binding activity estimated from scATAC; *ATAC_TFact.p_val_adj* = adjusted p-value from the differential activity analysis performed with Wilcoxon test; ATAC_TFact = 1 if the TF binding motifs are differentially accessible.

“Mouse.” and “macaque.” refer to differential expression analysis in the respective species, with *avg_logFC* = average log2-Fold change in expression; *pct.1* = % cells expressing the TF in the cell type; *pct.2* = % cells expressing the TF in the remaining cell types of the lineage; *p_val_adj* = adjusted p-value from the differential expression analysis performed with Wilcoxon test. Mouse orthologous gene is indicated at the “orthologous_gene_mouse” column.

**Supplementary Table 6. Extended_Table6.xlsx**

**Somatic cells summary for each scRNAseq sample.** All tables contain the number of somatic cell states from each library for (**A**) human female scRNAseq analysis (**B**) human male scRNAseq analysis (**C**) mouse female scRNAseq analysis (**D**) mouse male scRNAseq analysis.

**Supplementary Table 7. Extended_Table7.xlsx**

**TF cross-species multiomic analysis in female supporting cells.** Table containing the multiple TF measurements in human, mouse and macaque used to prioritise TFs relevant for female supporting cell differentiation. Statistics estimated with the one-sided Wilcoxon Rank Sum test implemented in the FindAllMarkers function in Seurat. Columns across table indicate:

*TF* = transcription factor; *cluster* = cell type; *N_agreement* = number of evidences where the TF is significant in the cell type; *ATAC_PWM* = PWM used to estimate motif enrichment; *RNA_DEGs.avg_logFC* = average log2-Fold change in expression; *RNA_DEGs.pct.1* = % cells expressing the TF in the cell type; *RNA_DEGs.pct.2* = % cells expressing the TF in the remaining cell types of the lineage; *RNA_DEGs.p_val_adj* = adjusted p-value from the differential expression analysis performed with Wilcoxon test; *RNA_DEGs* = 1 if it is a differentially expressed gene; *RNA_TFact.avg_logFC* = average log2-Fold change in activity estimated from Dorothea regulons; *RNA_TFact.p_val_adj* = adjusted p-value from the differential activity analysis performed with Wilcoxon test; *RNA_TFact.Dorothea_score* = Dorothe score for the TF; RNA_TFact = 1 if it is a differentially activated TF; ATAC_TFact.avg_logFC = average log2-Fold change in binding activity estimated from scATAC; *ATAC_TFact.p_val_adj* = adjusted p-value from the differential activity analysis performed with Wilcoxon test; ATAC_TFact = 1 if the TF binding motifs are differentially accessible.

“Mouse.” and “macaque.” refer to differential expression analysis in the respective species, with *avg_logFC* = average log2-Fold change in expression; *pct.1* = % cells expressing the TF in the cell type; *pct.2* = % cells expressing the TF in the remaining cell types of the lineage; *p_val_adj* = adjusted p-value from the differential expression analysis performed with Wilcoxon test. Mouse orthologous gene is indicated at the “orthologous_gene_mouse” column.

**Supplementary Table 8. Extended_Table8.xlsx**

**CellPhoneDB interactions (germ-female supporting lineage).** Columns represent interaction cell pairs, rows represent the interactions. Table is binary, with 1 indicating that all the members of the interaction are expressed in at least 10% cells and at least one member is a DEGs with a log2-fold change above 0.02 and adjusted p-value < 0.001.

**Supplementary Table 9. Extended_Table9.xlsx**

**Immune cells summary for each scRNAseq sample.** (**A**) Number of immune cell states from each library for human scRNAseq analysis (**B**) Table containing results from the differential expression analysis in the gonadal immune cell types. (**C**) Table containing results from the differential expression analysis in the myeloid cell types across organs.

**Supplementary Table 10. Extended_Table10.xlsx**

**RNAscope probes.** Probes used for smFISH.

**Supplementary Table 11. Extended_Table11.xlsx**

**CellphoneDB v4 database and CellSign.** (**A**) New manually curated L-Rinteractions. (**B**) Complexes involved in the novel CellphoneDB interactions. (**C**) CellSign: new manually curated Receptor-TF interactions.
